# Structure prediction of honey bee vitellogenin: a multi‐domain protein important for insect immunity

**DOI:** 10.1002/2211-5463.13316

**Published:** 2021-10-31

**Authors:** Vilde Leipart, Mateu Montserrat‐Canals, Eva S. Cunha, Hartmut Luecke, Elías Herrero‐Galán, Øyvind Halskau, Gro V. Amdam

**Affiliations:** ^1^ Faculty of Environmental Sciences and Natural Resource Management Norwegian University of Life Sciences Aas Norway; ^2^ Norwegian Center for Molecular Medicine University of Oslo Norway; ^3^ Department of Physiology and Biophysics University of California Irvine CA USA; ^4^ Department of Structure of Macromolecules Centro Nacional de Biotecnología (CNB‐CSIC) Madrid Spain; ^5^ Department of Biological Sciences University of Bergen Norway; ^6^ School of Life Sciences Arizona State University Tempe AZ United States; ^7^ Present address: E. Herrero-Galan, Molecular Mechanics of the Cardiovascular System Cell and Developmental Biology Area Centro Nacional de Investigaciones Cardiovasculares (CNIC) Instituto de Salud Carlos III C/ Melchor Fernández Almagro Madrid Spain

**Keywords:** homology modeling, honey bee vitellogenin, rigid‐body fitting, von Willebrand factor domain

## Abstract

Vitellogenin (Vg) has been implicated as a central protein in the immunity of egg‐laying animals. Studies on a diverse set of species suggest that Vg supports health and longevity through binding to pathogens. Specific studies of honey bees (*Apis mellifera*) further indicate that the *vitellogenin* (*vg*) gene undergoes selection driven by local pathogen pressures. Determining the complete 3D structure of full‐length Vg (flVg) protein will provide insights regarding the structure–function relationships underlying allelic variation. Honey bee Vg has been described in terms of function, and two subdomains have been structurally described, while information about the other domains is lacking. Here, we present a structure prediction, restrained by experimental data, of flVg from honey bees. To achieve this, we performed homology modeling and used AlphaFold before using a negative‐stain electron microscopy map to restrict, orient, and validate our 3D model. Our approach identified a highly conserved Ca^2+^‐ion‐binding site in a von Willebrand factor domain that might be central to Vg function. Thereafter, we used rigid‐body fitting to predict the relative position of high‐resolution domains in a flVg model. This mapping represents the first experimentally validated full‐length protein model of a Vg protein and is thus relevant for understanding Vg in numerous species. Our results are also specifically relevant to honey bee health, which is a topic of global concern due to rapidly declining pollinator numbers.

## Graphical abstract

We describe, for the first time, a full‐length protein structure of honey bee vitellogenin (Vg) using homology modeling, AlphaFold, and a negative stain electron microscopy map. Revealing the oligomeric arrangement and composition of the functional domains around the binding cavities, brings us closer to understanding the molecular mechanisms for Vg's functional role in insect immunity.

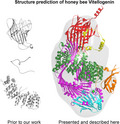

**Abbreviations**
BN‐PAGE: blue native polyacrylamide gel electrophoresis
CCS: cross‐correlation score
DAMPs: damage‐associated molecular patterns
DUF1943/1944: domain of unknown function 1943/1944
EM: electron microscopy
fbVg: fat body Vg
flVg: full‐length Vg
LC: lower cavity
MSA: multiple sequence alignment
MTP: microsomal triglyceride transfer protein
ND: N‐terminal domain
PAMPs: pathogen‐associated molecular patterns
QMEAN: Qualitative Model Energy Analysis
SEC: size exclusion chromatography
spdbv
: Swiss‐PdbViewer
UC: upper cavity
VADAR: volume, area, dihedral, angle reporter
Vg: vitellogenin
vWF: von Willebrand factor
Ω: gap region

Vitellogenin (Vg) belongs to an ancient and phylogenetically broad protein family called large lipid transfer proteins [1]. In most egg‐laying animals, Vg contributes to oogenesis by providing lipids. Over the last 20 years, studies of several species have demonstrated additional functions of this superfamily in health and behavior [2]. Many animals with one or more *vg* genes are commercially important, and this has incentivized analyses of reproductive and immune traits in which Vg is likely to play a role. Effects of Vg on host immunity have been studied in animals as diverse as bees and fishes [3, 4]. For example, Vg recognizes gram‐positive bacteria (i.e., *Staphylococcus aureus*, *Micrococcus luteus,* and *Bacillus subtilis*) and gram‐negative bacteria (i.e., *Escherichia coli* and *Vibrio anguillarum*) in nonbilaterian coral (*Euphyllia* *ancora*) and zebrafish (*Danio rerio*) [5, 6]. These studies also show that Vg recognizes general bacterial and fungal pathogen‐associated molecular patterns (PAMPs). Antimicrobial activity was not detected in these studies, but the interaction promotes apoptosis. Zhang et al. [4] suggest that Vg in zebrafish functions as an inflammatory acute‐phase protein leading to elimination of pathogens. This finding also applies to honey bees (*Apis mellifera*) where Vg appears to have similar immunological binding properties [7]. In addition, the Vg molecule of honey bees recognizes damage‐associated molecular patterns (DAMPs) [3] and displays antioxidant activity [8, 9, 10].

The honey bee is one of the best studied species in terms of the diverse roles of Vg [8, 11, 12]. For example, this animal was used to show that via their eggs, females can protect their offspring against diseases using a Vg‐mediated transfer mechanism: Fragments of bacterial cell walls (immune elicitors) are recognized by Vg and carried out to the honey bee eggs during oogenesis [7, 13]. This phenomenon of trans‐generational immune priming without the use of antibody‐based (i.e., acquired) immunity was first detected a decade ago [14]. However, the underlying mechanisms were not understood before Vg was proposed as a causal element [7]. The availability of the genomic sequence and some functional genetic technologies in honey bees have also enabled studies of Vg's role in behavior [8, 15], and such findings have been extended to ants, cockroaches, and mosquitos [16, 17, 18]. Honey bees are globally available due to apiculture and can be obtained in large numbers at low costs. Therefore, honey bees provide a practical and useful model for investigating the structure–function relationship of Vg.

In most egg‐laying animals, Vg consists of three conserved domains: The N‐terminal domain (ND), a domain of unknown function 1943 (DUF1943) and the von Willebrand factor (vWF) type D domain (Fig. S1). In honey bees, the ND is further subcategorized into two structural subdomains, the β‐barrel and the α‐helical domains, with a highly disordered polyserine region linking these two domains [19] (Fig. S1A). Circulating Vg in the hemolymph of honey bees has a molecular mass of approximately 180 kDa. Vg is cleaved into a 40 and a 150 kDa fragment in the abdominal fat body tissue, the main site for Vg synthesis and storage, and the polyserine linker has been identified as the cleavage site [19]. During investigation of pathogen recognition of Vg in honey bees, the full‐length hemolymph Vg (flVg) and the 150 kDa fat body Vg (fbVg) subunit, together with a recombinant peptide of the α‐helical domain, were shown to recognize dead and damaged cells [3]. The authors suggest that the heavily positively charged α‐helical domain is the main contributor to pathogen recognition. The same study also includes a recombinant peptide of vWF, but this synthetic domain did not show similar binding activity. Studies in fishes and one coral species confirm that the ND can recognize PAMPs and DAMPs but also show that the DUF1943 and vWF can contribute to pathogen recognition [5, 6]. Taken together, these findings indicate that Vg may have multiple pathogen‐recognizing domains.

In vertebrates and invertebrates, the three main structural domains of Vg are highly conserved at the structural level [5] despite a low nucleic acid sequence similarity [1]. This conservation indicates that the main features of the Vg amino acid sequence are maintained by natural selection. At the level of nucleic acids, the β‐barrel subdomain is the most conserved region of the honey bee *vg* gene, while the presumed lipid‐binding region (α‐helical domain and DUF1943) undergoes positive selection [20]. In a previous study, five residue positions were identified as candidates of functional polymorphisms (marked in Fig. S1A). Local pathogen pressure can be a significant selective force [21, 22, 23], and several studies suggest that Vg structure adapts to more efficiently recognize such local threats [7, 12]. This hypothesis relies on structure–function relationships that are not fully understood. In fact, there is no complete and detailed structure of the full‐length Vg (flVg) protein in any bee, insect, coral, or modern fish species. The only experimentally solved structure is that of lamprey (*Ichthyomyzon unicuspis*) Vg (PDB ID: 1LSH [24]), which consists only of the lipovitellin light and heavy chain (ca. 76% of the sequence is crystallized; Fig. S1B). Using this information as a resource, the conserved N‐terminal subdomains (β‐barrel and α‐helical) in honey bees were described using homology modeling [3, 25] with lamprey Vg as a template. This approach has not been extended to the less conserved DUF1943 domain that is also present in lamprey. The vWF homologous domain, β‐Component, is absent from the lamprey crystallographic structure, which eliminates lamprey as a possible template for homology modeling of the vWF domain in other species like honey bees.

Solving the structure of Vg in more species can increase our understanding of ligand interactions and provide important insights into structure–function relationships. However, even in otherwise well‐studied species like honey bees, this centrally important information on the DUF1943 and vWF domain is lacking.

Fortunately, the number of experimentally solved protein structures is growing, and the computational modeling software is becoming more powerful. For example, a crystallographic protein structure of the D’D3 assembly in human vWF protein was resolved in 2019 [26], and the VWD3 domain in this assembly has a pairwise sequence identity slightly above 20% to the honey bee domain, which is sufficient to be used as a template during homology modeling.

In this study, we make progress in describing the structure and interpreting the function of the vWF domain in honey bees. In addition, we compile results from template‐based, deep learning modeling methods, and the ground‐breaking neural network‐based algorithm, AlphaFold [27], to present, for the first time, a full‐length model for an invertebrate Vg. We combine this new information with published data to begin to elucidate the domain assembly of flVg. Our findings suggest that vWF contributes to the structural organization and has a previously undescribed and valuable function in the protein. This study contributes to the understanding of a protein that is central to life in many animal species.

## Materials and methods

### Identification of templates

The full‐length honey bee Vg sequence (UniProt ID: Q868N5) was inputted to the HHpred [28] server with default settings, which included ‘PDB_mmCIF70_23_Jul’ as the target database. HHpred returned 250 hits. Each hit was evaluated based on the sequence identity. For the vWF domain, the structural template was verified by performing a blast of honey bee Vg (UniProt ID: Q868N5) against the UniProtKB. The target database was restricted to only include UniProt sequences having a PDB ID. The query was run with default settings (*e*‐threshold: 10, matrix: auto, filtering: none, gapped: yes, hits: 1000). This blast returned 26 hits, and hits from regions already satisfactorily modeled in earlier work were ignored. The remaining hits included the VWF_HUMAN (UniProt ID: P04275, *e*‐value 7.2e‐1, and 25.0% sequence identity). Residues 1453–1612 of the vWF domain in Vg were aligned to residues 864–1013 of vWF, *Homo sapiens*. These residues correspond to the WD3 domain in the D’D3 assembly in the human vWF protein.

### Structural alignment and homology modeling of the von Willebrand factor domain

Both the target and template sequence are part of two larger assemblies, each comprising 4 and 12 domains, respectively. To identify the correct start and end points of the structural alignments, 16 alignments with different sequence lengths were performed. The highest sequence identity (26.3%) was obtained by aligning residues 1440–1634 (target) with residues 836–1031 (template) using the Emboss Needle pairwise alignment tool [29, 30], with default settings (Table S1). To ensure that the functional and important regions were aligned correctly, the pairwise alignment was supplemented with a multiple sequence alignment (MSA). The MSA was executed using blast and representative Vg sequences from a wider selection of 16 species [3] (Table S2). To ensure a correct alignment of the full‐length vWF *H. sapiens* in the MSA and not cause confusion among the four VWD modules in the protein, we referenced the alignment of the modules in the D assemblies from Dong et al. [26] (Fig. 2). The pairwise alignment was altered so that gaps were in the same positions as in the low‐conserved regions of the MSA. The highly conserved residues were correctly aligned and were not altered. To avoid gaps in secondary structures or binding sites, the secondary structure annotations from template 6N29 were added to the alignment.

The homology model was interactively built using Swiss‐PdbViewer [31] (spdbv; v. 4.1.0), a recommended approach when building target models with low sequence identity to the template [32]. To initiate the modeling project, the raw sequence (Q868N5) was fitted onto the 3D coordinates of the template (PDB ID: 6N29). Backbone building was performed automatically after editing the alignment as described above. *Ab initio* loop building was performed to ligate breaks in the backbone caused by gaps in the alignment (insertions/deletions). The loop option with the lowest clash and energy scores was chosen in all cases. In this way, nine loops were inserted into the model (Table S3), leaving three unsolved regions (residues 1494–1504, 1515–1522, and 1537–1541) missing in the model. *Ab initio* and database loop building attempts failed to produce a reasonable output for these three 8–11 residue‐long gaps. Side chain conformations of target residues aligned to residues with dissimilar characteristics in the template were identified by detecting clashes and rearranged into the most optimal rotamer option. Rotamer libraries of the most observed orientations for side chains are included in the program. The entire model was energy minimized through a partial implementation of the gromos96 force field [33] integrated in the spdbv software.

### Quality control of the von Willebrand factor homology model

Quality control was performed on the model to determine whether the structural features are consistent with the physiochemical rules. Stereochemical consistency was evaluated residue‐by‐residue using procheck [34]. Global and local quality estimates were performed using the Qualitative Model Energy Analysis (QMEAN) server [35], powered by SWISS‐MODEL. The QMEAN output *Z*‐score compares the query to similar values based on X‐ray structures. VADAR (v. 1.8) [36] assesses the 3D profile, stereo/packing, accessible surface and residue volume. Based on these quality assessments, manual editing was applied to the residues listed in Table S4. The final model was deposited to ModelArchive and can be accessed at: https://modelarchive.org/doi/10.5452/ma‐sfueo (access code: okHs98Pcl2).

The Ca^2+^‐ion was copied from the template to the target model, and the contacts to the binding residues were verified to be reasonable in pymol (v. 2.2.2) [37]. All illustrations of the model were made in pymol.

### Full‐length structure prediction of honey bee vitellogenin

The alignments from HHpred with the highest sequence identity were selected and forwarded to the implemented modeling software modeller [38]. Models 1–8 were built using the query sequences listed in Table 1. All models were built using default settings. A full‐length prediction was also built using the raptorx web server [39] with the full‐length honey bee Vg sequence (UniProt ID: Q868N5) as input, which generated a structure consisting of six domains, each built using one to five templates or template‐free modeling (Table S7 and Fig. S7). The models were visualized with the program pymol and aligned, and the final model was assembled and built here.

**Table 1:** Structure predictions generated by modeller and raptorx. The table presents all the models generated using modeller and raptorx (Figs S6 and S7) and lists the region of the amino acid sequence (aa seq.) that has been modeled and which domain it represents. The template used for the model (protein name, species, and PDB ID) and the sequence identity are listed. For Model 9, several templates have been used to generate the full‐length model.

Model	Honey bee Vg aa seq.	Honey bee Vg domain	Template	Seq. iden. (%)
1	21–1059	ND and DUF1943	Lamprey Vg (PDB ID: 1LSH_A)	16
2	1190–1515	Undetermined and partly vWF	Lamprey Vg (PDB ID: 1LSH_B)	15
3	1442–1632	vWF	Human vWF (PDB ID: 6N29)	22
4	21–323	β‐barrel	Lamprey Vg (PDB ID: 1LSH_A)	19
5	324–360	Polyserine linker	Honey bee Vg (PDB ID: 2ILC)	97
6	361–756	α‐helical	Lamprey Vg (PDB ID: 1LSH_A)	19
7	760–1059	DUF1943	Human MTP (PDB ID: 6I7S)	13
8	760–1059	DUF1943	Lamprey Vg (PDB ID: 1LSH_A)	11
9	1–1770	Full‐length Vg	PDB ID: 1LSH_A, 1LSH_B, 6RBF_A, 3WJB_A, 4YU8_A, 4JPH_A, 5BPA, 4NT5_A and 2KD3_A	12, 21, 8, 6, 5, 9, 10, 14 and 7

To run AlphaFold v2.0 ([27], see Jumper et al. (2021) supplementary material for detailed description of the method), a P3.2xlagre instance was provisioned from AWS EC2, using the Deep Learning AMI (Ubuntu 18.04) Version 48.0 and a 300 GB disk. Additionally, a 4TB gp3 EBS volume, with 400 MB·s^−1^ of throughput and 3000 IOPS, was provisioned and mounted on the machine. The step‐by‐step guide (README.md, https://github.com/deepmind/alphafold) was followed for setting up and running AlphaFold using Docker. Dependencies that were not included in the AMI were installed manually using the apt package manager. The input sequence was UniProt ID: Q868N5, and AlphaFold was run with the full_dbs preset. Model parameters, downloaded databases, and the output files were stored on the 4TB EBS volume. The run resulted in five models, ranked by average plDDT (Fig. S8B,C). The PDB‐file of the top ranked model is included in Appendix S2.

### Rigid‐body fitting into the electron microscopy map

The high‐resolution full‐length model and separate chains, in addition to two previously published homology models [3, 25] and lamprey Vg (PDB ID: 1LSH) [24], were fitted into the low‐resolution negative‐stain electron microscopy (EM) map (Fig. S9, EMDB‐22113, deposited) without direct human intervention by using the PowerFit webserver [40, 41] and the ADP_EM plugin in chimera [42]. In both methods, the resolution was set to 27 Å based on the Fourier shell correlation curve (Fig. S9C), and for PowerFit, the rotational sampling interval parameter was set to 5.00. The PowerFit algorithm uses the cross‐correlation between the EM map and the structure to be fitted to search for optimal fits. Output was provided as the structural model's orientation with a corresponding goodness of fit score. ADP_EM works similarly, but is optimized for low‐resolution density maps. The fits were imported to the program ucsf chimera (v. 1.14) [43] to optimize them using the volume data ‘Fit‐in‐map’ function. This function calculates a correlation score and an average map value both based on map grid points, but the former calculates overlap, while the latter only focuses on the atoms inside the map. In addition, the number of atoms outside the contour is shown. The setting was left as default, but the resolution of 27 Å was inputted. All resulting scores from both software systems are presented in Tables S5 and S6.

chimera and pymol were also used to generate the figures of the fits and apply a hydrophobicity scale [44]. The final assembly was imported to pymol, where it was aligned to lamprey Vg (PDB ID: 1LSH). The generate symmetry function in pymol was used to produce the dimer formation presented by Anderson et al. [53] of lamprey Vg and aligned the final assembly to this structure to present the dimer of honey bee Vg (Fig. 4E). The conserved residues creating polar contacts in honey bee Vg were identified using the MSA produced by modeller (not shown). The distances of polar contacts were measured in pymol.

### Purification of vitellogenin from honey bees

To obtain purified Vg, we collected 1–10 µL honey bee hemolymph in a 1 : 10 dilution in 0.5 m Tris/HCl pH 7.6, using BD needles (30 G) as described earlier [45]. The dilution was filtered using a 0.2 µm syringe filter. Vg was purified from honey bee hemolymph with ion‐exchange chromatography using a HiTrap Q FF 1 mL column 0.5 m Tris/HCl as the sample buffer and 0.5 m Tris/HCl with 0.45 m NaCl as the elution buffer. 400–450 µL diluted hemolymph was manually injected and Vg eluted at a conductivity of 15–22 mS·cm^−1^. All fractions from this peak were collected, pooled and concentrated using an Amicon^®^ Ultracel 100 kDa membrane centrifuge filter (Merck KGaA, Darmstadt, Germany). The fraction purity was verified by running SDS/PAGE, which contained only one band of the correct size (~ 180 kDa). The protein concentration was measured with Qubit.

### Native gel and size exclusion chromatography

Blue native polyacrylamide gel electrophoresis (BN‐PAGE) was performed at 4 °C in precast 3–12% acrylamide gels (Invitrogen, Waltham, MA, USA) for 2 h at a constant voltage of 150 V. The NativePAGE Novex Bis‐Tris Gel System (Life Technologies, Carlsbad, CA, USA) protocol was used both for sample and buffer preparation, and Native‐PAGE Running Buffer (1×) and the Dark Blue Cathode Buffer (0.4% Coomassie G‐250) were used. Size exclusion chromatography (SEC) was performed of Vg in a Superose 6 Increase 3.2/300 column (GE Healthcare, Chicago, IL, USA) at 4 °C equilibrated with a buffer containing 50 mm Tris pH 7.6 and 225 mm NaCl. The SEC was run on an ÄKTA Pure 25 system (GE Healthcare) in micro configuration that allows the use of very small sample volumes. This modification prevents dilution of the sample by effectively reducing the internal volume since it bypasses the multicolumn valve and the pH flow cell and has a shorter path length between the injection valve and the UV monitor. We injected 50 µL of sample (0.26 mg·mL^−1^) and manually collected fractions directly from the outlet of the UV monitor.

## Results

### Template search

**Fig. 1:**
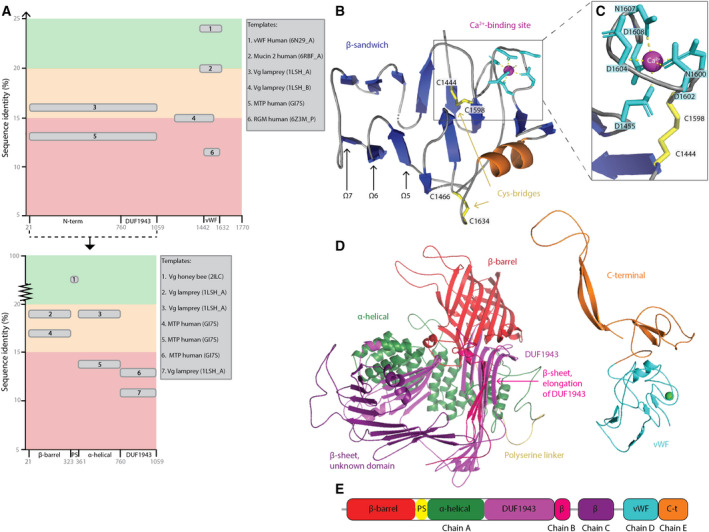
Structure prediction of honey bee vitellogenin. (A) A graphical illustration of the identified templates using HHpred. On both graphs, the amino acid sequence of honey bee Vg is on the *x*‐axis (with the subdomains and domains labeled), and the percentage of sequence identity to the templates is on the *y*‐axis. The first graph displays all the templates (gray rounded edge boxes) identified when inputting the full‐length sequence of honey bee Vg, while the second shows the templates identified when inputting only the sequence of the separate subdomains. The background colors on both graphs illustrate whether the sequence identity is below 15% (red), between 15% and 20% (orange) or above 20% (green). The templates are numbered according to the sequence identity (highest to lowest), and the protein name, species, and PDB ID are noted in the two large gray boxes. (B) Homology model of vWF: The β‐sandwich is on the left side while the Ca^2+^‐segment is on the right side. The Cys‐bridges connecting the two segments are shown as yellow sticks and arrows. The β‐strands, α‐helix and loops are colored blue, orange, and gray, respectively, and the positions of Ω5–7 are labeled with black arrows. The Ca^2+^‐binding residues are shown as cyan sticks, and the Ca^2+^‐ion is shown as a pink sphere. (C) Close‐up of the Ca^2+^‐binding site. The coloring scheme is the same as in panel B. All Ca^2+^‐binding residues (D1455, N1600, D1602, D1604, N1607 and D1608) and one of the Cys‐bridges (C1598 and C1444) are labeled, and this demonstrates how D1455 from the β‐sandwich interacts with the Ca^2+^‐ion. (D) The full‐length homology model compiled from several models with different templates. The subdomains and domains are colored as follows: the β‐barrel subdomain (red), the polyserine linker (yellow), the α‐helical subdomain (forest green), the DUF1943 domain (magenta), elongation of the DUF1943 domain (hot pink), the undetermined structural region (purple), the vWF domain (cyan), and the C‐terminal region (orange). (E) A 2D illustration of the chains A to E, used when preforming rigid‐body fitting of the homology model.

Increased insight into the tertiary structure of Vg's domains is beneficial to our understanding of how Vg contributes to honey bee immunity. To build a full‐length structure prediction of honey bee Vg, we first identified potential templates using HHpred [28] (Fig. 1A) with the complete amino acid sequence as input. HHpred indicated that two templates are available for building the ND and DUF1943 domain, one for an undetermined region (residue 1190–1442), and three for the vWF domain. Except for Template 1 (PDB ID: 6N29_A), the sequence identities fall below 20%. By dividing the query sequence into known subdomains and domain boundaries and repeating the search, we generated more specific alignments. The top two ND subdomain templates increased their sequence identities to 19%. In contrast, the DUF1943 was demonstrated to be more distinct compared to human microsomal triglyceride transfer protein (MTP) and lamprey Vg, having sequence identities of only 13% and 11%, respectively.

### Homology modeling of the von Willebrand factor domain

Among the three highly conserved domains, the vWF is a major unknown piece in the structural puzzle of Vg. Our initial search discovered a recently published and promising template for this domain, which we confirmed using blast [46]. The WD3 domain in the D’D3 assembly of the vWF protein of *H. sapiens* has a sequence identity of the pairwise alignment of 24.1%, which is slightly below the suggested threshold (25%) for creating a reliable homology model [47]. In other words, a pairwise alignment may not be enough to identify gaps and robustly conserved amino acids. We therefore conducted a MSA to confirm gaps and alignment of conserved and domain‐defining residues across 12 species, including representative insects, nematodes and mammals. The MSA and the final structural alignment are presented in Fig. S2.

A visual inspection of the structural alignment revealed some interesting aspects. In the almost 200 amino acid‐long alignment, the first 40 residues and the last 80 residues are well conserved. In the less conserved regions, four larger gap regions (Ω) have been introduced (Ω4–7). Ω4 is also missing in all species containing the vWF protein based on the MSA, while downstream Ω5 and Ω7 are conserved in most of the species containing the Vg protein (Fig. S2A). Ω6 seems to be included in all species but is missing in the VWD3, a cysteine‐rich domain that forms four intrachain disulfide bridges and two interchain disulfide bridges. The interchain bridges stabilize dimerization of VWD domains in the human vWF protein as opposed to the intrachain bridges formed between cysteine residues inside a single VWD domain. The interchain bridging cysteine residues are not included in the target sequence, and based on the MSA, they are also not conserved in the template domain. However, the eight intrachain bridging cysteine residues are included in the template. Four of these are conserved in the target (C1444, C1466, C1598, and C1634; Fig. 1B). The VWD3 domain also contains a Ca^2+^‐binding site experimentally known from the structural template with key residues also present in the target sequence [26]. We recognize this as a class II calcium binding site because the coordinating residues, as well as the neighboring residues, make up two short regions [48] (r. 1453–1456 and r. 1596–1609; Fig. S2) that are well conserved among all species in the MSA. This indicates an essential site for function and/or stability of the domain. We conclude that the significant regions for domain function or stability, the intrachain disulfide bonds, as well as the Ca^2+^‐binding residues, are conserved and correctly aligned. We also conclude that the MSA was able to identify robustly conserved features of Vg, and we therefore proceeded with interactive homology modeling using the structural alignment provided by the MSA (Fig. S2B).

The amino acid sequence of the target was fitted onto the three‐dimensional coordinates of the template using the structural alignment. Breaks in the backbone were ligated using loop building, and the side chains of nonconserved residues were rearranged to the most optimal rotamer orientation, reducing the number of steric clashes. Finally, we performed energy minimization to release local backbone strain and electron density clashes. The overall quality of the target model was validated using several software tools. To account for sequential errors, we also included the quality scores of the template (Figs S3 and S4). Based on the results, the backbone phi and psi angles of 14 residues, detected as outliers by Ramachandran analysis (Fig. S3C) [49], and rotamers of 19 residues, detected by procheck, were manually edited (Table S4). The main limiting factor for the quality metrics of the model were the errors already listed as well as the presence of the longer gap regions. It was not possible to include Ω5–7 in the model because this creates a region with too many unfavorable interactions and torsion angles. However, these regions exhibit low conservation (Fig. S2). The local quality estimate by SWISS‐MODEL (Fig. S3B) shows that the middle region is of lower quality relative to the first and last missing regions. The Ca^2+^‐binding residues and intrachain disulfide bonds are in higher‐quality regions. The procheck summary shows that the main difference between the target and template models originates from the calculated stereochemical parameters (geometry, bad contacts and bond length and angles; Fig. S3A). The residue‐by‐residue list produced by procheck (Fig. S4E) identified residues deviating from the ideal values. However, these residues were altered during loop building, often resulting in an unfavorable orientation for the chosen residues [50]. We conclude that key structural features of the target are modeled correctly except for the low‐quality middle region that contains residues with stereochemical parameters deviating from the ideal values. The homology modeling approach used has a proven track record of producing models of sufficient quality when facing similar challenges [51]. We demonstrated this by comparing our model to an automatically produced model by modeller. We find that in our model, the local quality is better in the regions of low conservation (Fig. S3B), and the global quality is higher (Fig. S4A–C). For the conserved region, our interactive modeling approach achieves a better result by including C1634, which creates an intrachain disulfide bond, two additional β‐strands and a more appropriate rotamer option for the Ca^2+^‐binding residue N1607 (Fig. S5).

We are thus for the first time able to present a detailed structural model of the vWF domain of honey bee Vg. The structure can be understood as two segments: one consisting of 11 antiparallel β‐strands organized into a β‐sandwich while the other is comprised of the Ca^2+^‐binding site, a short α‐helix, and three short β‐strands (Fig. 1B,C). Connecting the two segments are the two intrachain disulfide bonds. The two segments are also connected through the Ca^2+^‐binding site via the interaction of residue D1455 (Fig. 1C). The Ca^2+^‐binding residues are in loop regions (i.e., normally flexible regions), but we suggest that binding of a Ca^2+^‐ion might confer stability to this region. The Ca^2+^‐binding segment of the domain exhibits higher quality than the antiparallel β‐sandwich. Despite the lower quality, the residues in the secondary structure elements exhibit a higher local quality score compared to the residues in the loop regions (Fig. S3B). We conclude that the β‐strands are organized in a sterically reasonable manner, while the loop regions are most likely not described accurately.

### Full‐length structure prediction of honey bee vitellogenin

We performed template‐based prediction of the remaining domains of honey bee Vg using the integrated modeller software in HHpred. We generated eight models using different sections of the honey bee Vg amino acid sequence as input (Table 1). By aligning the predicted models covering the same domains (Fig. S6), we observed that the general fold is the same except for models describing DUF1943 (Models 1, 7, and 8; Fig. S6B). Using human MTP as a template returned a straight β‐sheet with fewer and longer β‐strands. In addition, we also used the deep learning modeling method raptorx to generate a full‐length and complete prediction (Fig. S7). The model is mostly based on nine different templates with sequence identity ranging from 5% to 21% but also includes regions resulting from deep learning predictions. The total model assembles all predicted domains like pearls on a string and cannot predict how they are organized relative to each other. However, the general fold of each model is consistent with the results from modeller (Fig. S6A–E). We built the final structure using Model 1 for residues 21–1059, Model 9 for residues 1060–1140, Model 2 for residues 1190–1408, the vWF homology model from Quality control of the von Willebrand factor homology model for residues 1440–1634 and Model 9 for residues 1635–1770. We selected these models based on whether their fold were consensus folds and removed the long, extending loop regions. The final model has 93.1% sequence coverage of honey bee Vg and includes the conserved domains (ND, DUF1943 and vWF) in addition to undetermined regions now structurally described for the first time for an invertebrate Vg (two β‐sheets downstream of DUF1943 and the C‐terminal region; Fig. 1D). Based on the compilation of models, the final prediction was divided into chains A (the ND), B (the β‐sheet from Model 9), C (the β‐sheet from Model 2), D (the vWF domain) and E (the C‐terminal region) as presented in Fig. 1E.

**Fig. 2:**
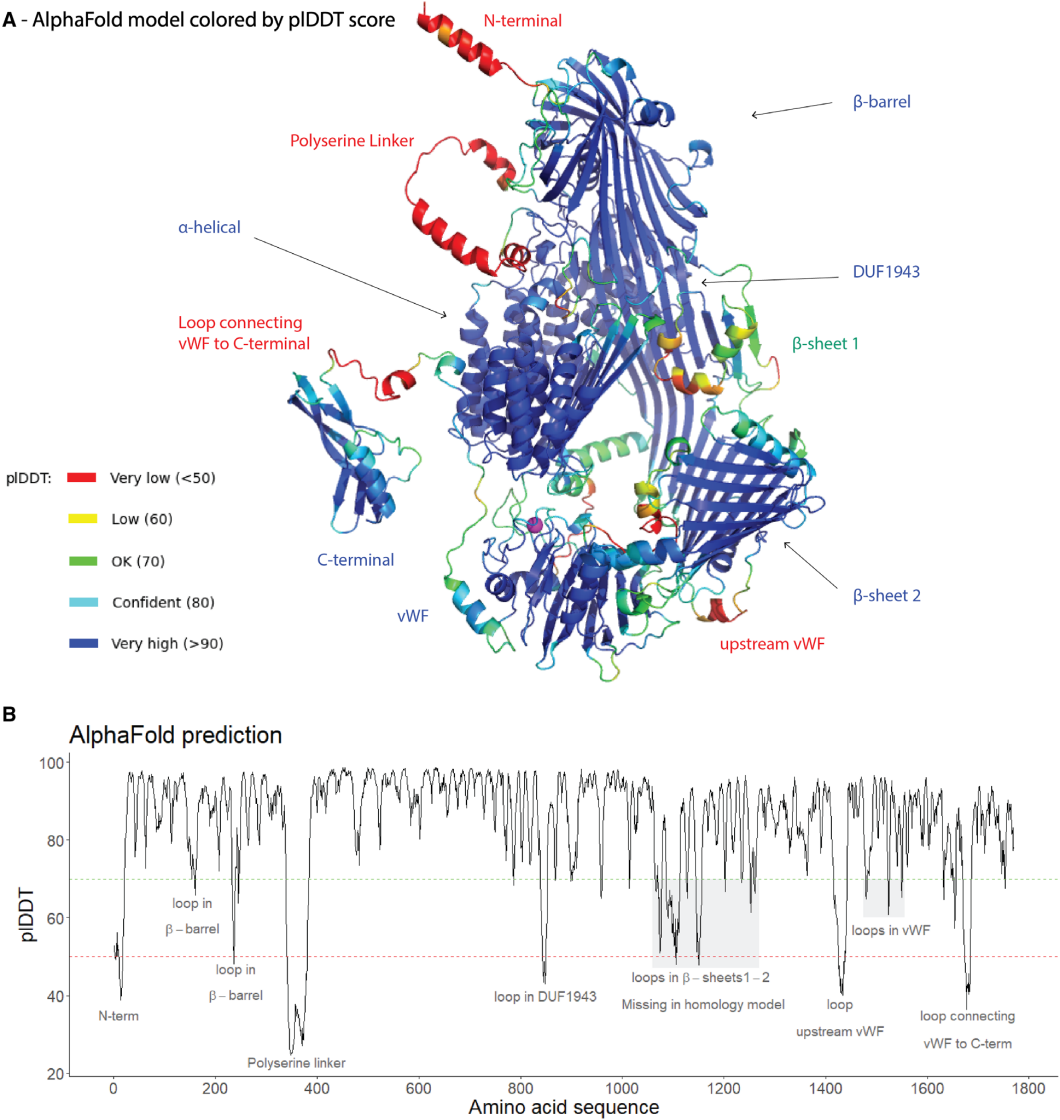
AlphaFold prediction. (A) The top ranked AlphaFold model is shown as cartoon, colored by the plDDT scale. The high scoring domains (β‐barrel, α‐helical, DUF1943, β‐sheet 2, vWF domain, and C‐terminal domain) are labeled in blue, while the medium confident region (β‐sheet 1) is labeled in green, and the low confident regions (N‐terminal, polyserine linker, the segment upstream and downstream of vWF domain) are labeled in red. The Ca^2+^‐ion is shown as a magenta sphere. (B) The plDDT score is plotted per residue for the top ranked AlphaFold model. Each region that scores below 70 (green dotted line) is labeled. The very low plDDT (< 50) is indicated with a red dotted line.

The very recent publication and code availability for AlphaFold v2.0 [27] enabled us to produce a structure prediction of honey bee Vg. The first step of the pipeline is to produce an MSA, and the resulting number of hits can indicate the prediction accuracy. The developers observe a decrease in prediction accuracy when the alignment depth falls below 30 sequences and an increase of accuracy until 100 sequences, where they observe a threshold effect [27]. The honey bee Vg MSA have an average of 1988 hits per residue (Fig. S8A), suggesting a high prediction quality. The resulting AlphaFold models had an average predicted local distance difference test (plDDT) ranging from 81.7692 to 84.5747 (Fig. S8B), which is a per‐residue estimate of confidence [27, 52]. The highest‐ranking model colored by the plDDT confidence scale (Fig. 2A) shows a generally confident backbone prediction of honey bee Vg. Some regions fall below 70, which the developers of AlphaFold state should be treated with caution, and these residues map to short loops in domains or longer flexible segments in‐between domains (Fig. 2B). The developers state that plDDT residue scores below 50 strongly indicate disorder which in our case is consistent with our knowledge of the protein. The very low scoring residues 341–380 (average pIDDT: 33.1242) map to the polyserine linker, which is known to be flexible and disordered [19]. Similar disorder is predicted for the N‐terminal signal peptide residues 1–17 (average plDDT: 47.8064) and the segments upstream and downstream of the vWF domain, residue 1425–1437 and 1674–1684 (average plDDT: 44.5930 and 42.9336), respectively. Aligning the top ranking AlphaFold predictions demonstrates a consistent fold for the confident regions and some inconsistency of the low‐confidence regions (Fig. S8C). The predicted disorder of residues 1674–1684 results in a variable positioning of the downstream C‐terminal region between the predictions, suggesting flexibility of the domain position.

The final homology model and the AlphaFold prediction agree on the fold of the stable domain (Fig. S8D). AlphaFold produces 3D coordinates for every atom in the protein, so the prediction takes up more space, compared to the homology model where there are missing atoms, particularly downstream of the DUF1943 domain (Fig. S8D). However, the overall consistency in both of our predictions confirms that our structural prediction is strong.

### Using PowerFit, ADP_EM, and Chimera to determine the domain assembly of full‐length vitellogenin

The full‐length models of Vg indicate the general fold of each domain. However, the domain assembly in the final homology model is speculative and derived from lamprey Vg and the deep learning method along with strong biases. To reduce these biases and provide some validation of the structural assembly, we performed rigid‐body fitting of our model to a low‐resolution EM map (Fig. S9, EMDB‐22113, deposited) of *in* *vivo*‐obtained honey bee Vg. The EM map reveals a rough overview of the surface and two distinct cavities, hereafter named top, base, left and right, upper cavity (UC) and lower cavity (LC) in reference to this specific orientation (Fig. S10A). Fitting of the complete homology model placed chains D and E consistently outside the contour map, while chains A to C did not take up all the available space inside it (Fig. S10C). This indicates incorrect domain assembly of chains D and E. Fitting of the raptorx structure gave similar results leaving chains C, D, and E outside the contour map, clearly demonstrating improper domain assembly (Fig. S10D). To avoid problems related to template‐based assembly, we fitted the chains individually. Chains A and D occupy somewhat separate parts of the contour, but chain A overlaps with chain C and partly chain B and E (Fig. S10E,F). These individual domain fits support the assembly of chain A to C in the predicted model and further suggest improper assembly of chains D and E. Keeping chains A to C united but chains D and E separate resulted in two alternative orientations (Fig. S11B,C) leaving out chain E, which is not compatible with either alternative (Fig. S10F). The first 68 residues of chain E were built using a template‐free method, while the last 58 residues were compiled from a multiple alignment of the last five templates (Table S7) ranging from 5% to 14% sequence identity. HHpred recognizes none of these templates. Faced with a speculative prediction and its incompatibility with the EM map, we removed the C‐terminal domain from the domain assembly. The resulting fits from two independent rigid‐body fitting methods (PowerFit [40, 41] and ADP_EM [42]) was optimized using chimera fit‐in‐map [43], producing correlation scores that could be compared directly (Figs S11A, S10B, and S12A). The highest scoring fit of chain A to C from ADP_EM is overlapping perfectly with the second‐best fit from PowerFit (Fig. S11B1), while the highest scoring fit of the same chains from PowerFit is agreeing with the relative orientation of the domains. The best fit from Powerfit is not overlapping, however, with the second‐best fit from ADP_EM (Fig. S11B2). The correlation score for the second ADP_EM fit is lower, and more atoms are outside the contour, compared to the other fits. Both alternatives are compatible with the ADP_EM and the PowerFit orientation of chain D (Fig. S11C). Secondary structure elements from the α‐helical subdomain and DUF1943 are protruding outside the contour for both alternatives. For alternative 2, the DUF1943 and additionally the β‐barrel subdomain are seemingly restricting access to both cavities (Fig. S11D).

**Fig. 3:**
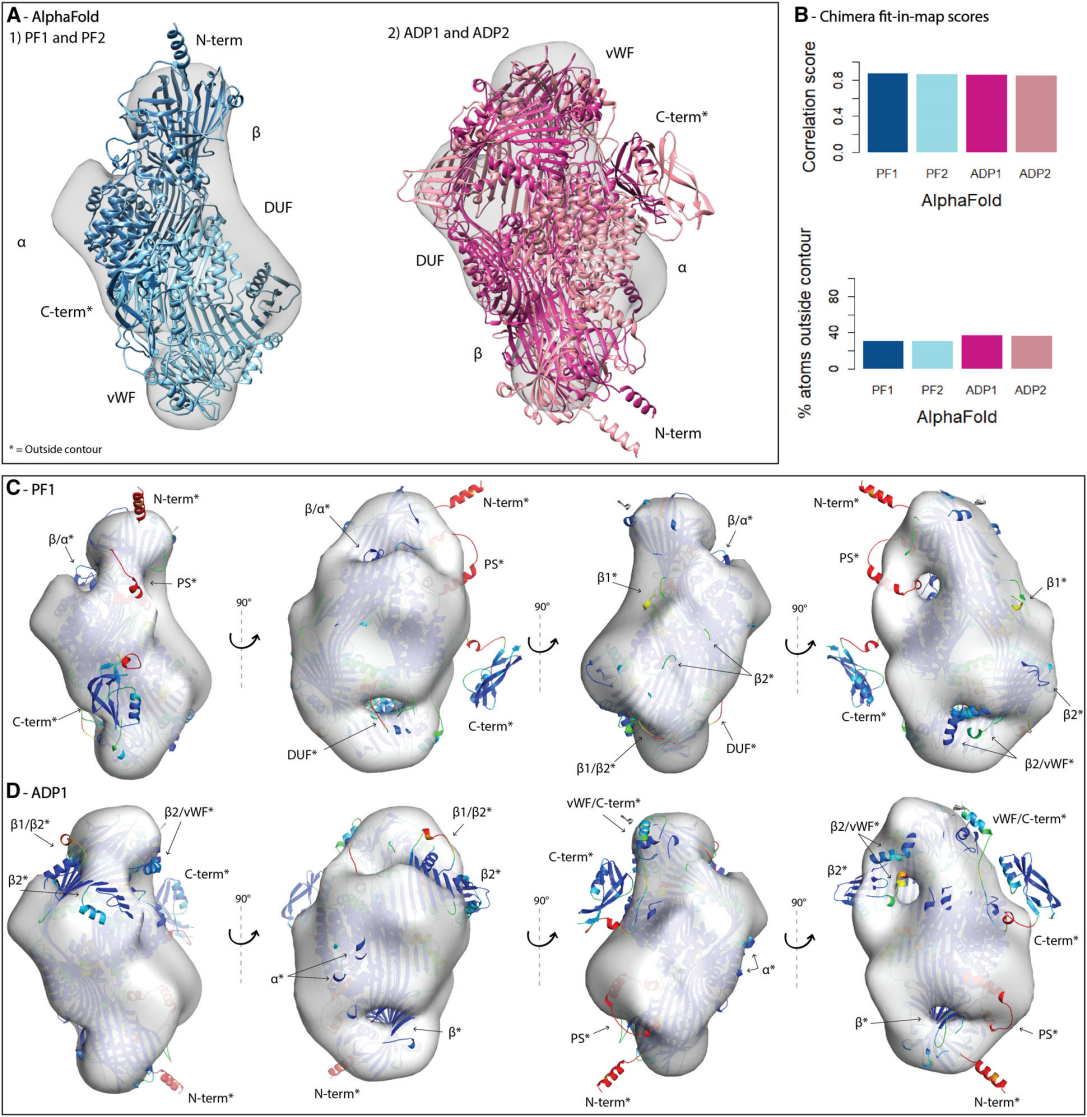
Rigid‐body fitting of AlphaFold. (A) The EM map are shown as a transparent surface, and the fits of AlphaFold from PowerFit (PF) and ADP_EM (ADP) are shown as cartoons and colored by method and scores (dark blue: PF1, light blue: PF2, dark pink: ADP1, light pink: ADP2). The N‐terminal (N‐term), β‐barrel (β), α‐helical (α), DUF1943 (DUF), vWF domain (vWF), and C‐terminal (C‐term) domains are labeled. (B) The correlation score and percent of atoms outside the contour calculated by chimera were plotted for each fit from PowerFit (PF, blue) and ADP_EM (ADP, pink), and ranked according to the correlation score (dark color: highest score, light color: second highest score). (C) The EM map and the highest ranked PowerFit fit of AlphaFold is shown in at four different angels, colored by plDDT score. The label is marked with ‘*’ if residues are outside the contour of the EM map and ‘/’ between domain labels indicate that the pointed to segment is in‐between domains. The polyserine linker and the two β‐sheets downstream of the DUF1943 domain are labeled PS, β1, and β2, respectively. (D) The EM map and the highest ranked ADP_EM fit of AlphaFold. The same coloring and labeling are used as in panel C.

To further investigate the two alternatives, we fitted previously generated homology models of the β‐barrel and α‐helical domains of honey bee Vg [3, 25] and the X‐ray structure of lamprey Vg (PDB ID: 1LSH [24]) to the EM map. The respective or homologous domains consistently fit in the two relative orientations and scored high values for both alternatives (Fig. S12). The β‐barrel and α‐helical domain supported alternative 1, while lamprey Vg favored alternative 2 according to the scores. The EM map is an *in vivo* representation of honey bee Vg, while the 1LSH structure is a distant homologue with 24% of the sequence missing in the crystal structure. The AlphaFold prediction with 100% sequence coverage serves as a far better representation of honey bee Vg. Fitting the top ranked AlphaFold prediction resulted in two different orientations by selecting the highest scoring fit from PowerFit and ADP_EM, respectively (Fig. 3A). The best fit from PowerFit has fewer atoms outside the contour and a higher correlation score, compared to the best fit from ADP_EM (Fig. 3B). The very low‐confidence fold of the N‐terminal signal peptide and the polyserine linker is protruding in both alternatives (Fig. 3C,D). In addition, smaller loops with a fold confidence ranging from low to intermediate are also protruding in both fits but these mismatches between model and contour map are more pronounced in the ADP_EM fit (Fig. 3D). The model cavities are restricted in the ADP_EM fit by the β‐barrel and a long β‐sheet which is the AlphaFold prediction of a more complete chain C, and these domains are confidently modeled. Both cavities in the PowerFit fit are also somewhat restricted by in‐between domains segments, which have a lower confidence fold. Taken together, the orientation represented by PowerFit is the best fit of the AlphaFold prediction. This orientation also conforms to the best fits of individual domains: chain A to C (Fig. S11B2), chain D (Fig. S11C, PF1), β‐barrel (Fig. S12B, ADP2), α‐helical (Fig. S12C, PF2 and ADP1) and lamprey Vg (Fig. S12D, PF1 and ADP1). This further supports the PowerFit orientation of the AlphaFold prediction, but now with a more optimized fit. Using the full‐length sequence representation results in a structure which fills more of the density space while keeping the percentage of protruding atoms low and the correlation score high. This suggests that the domain assembly in the AlphaFold prediction is an accurate representation of honey bee Vg.

**Fig. 4:**
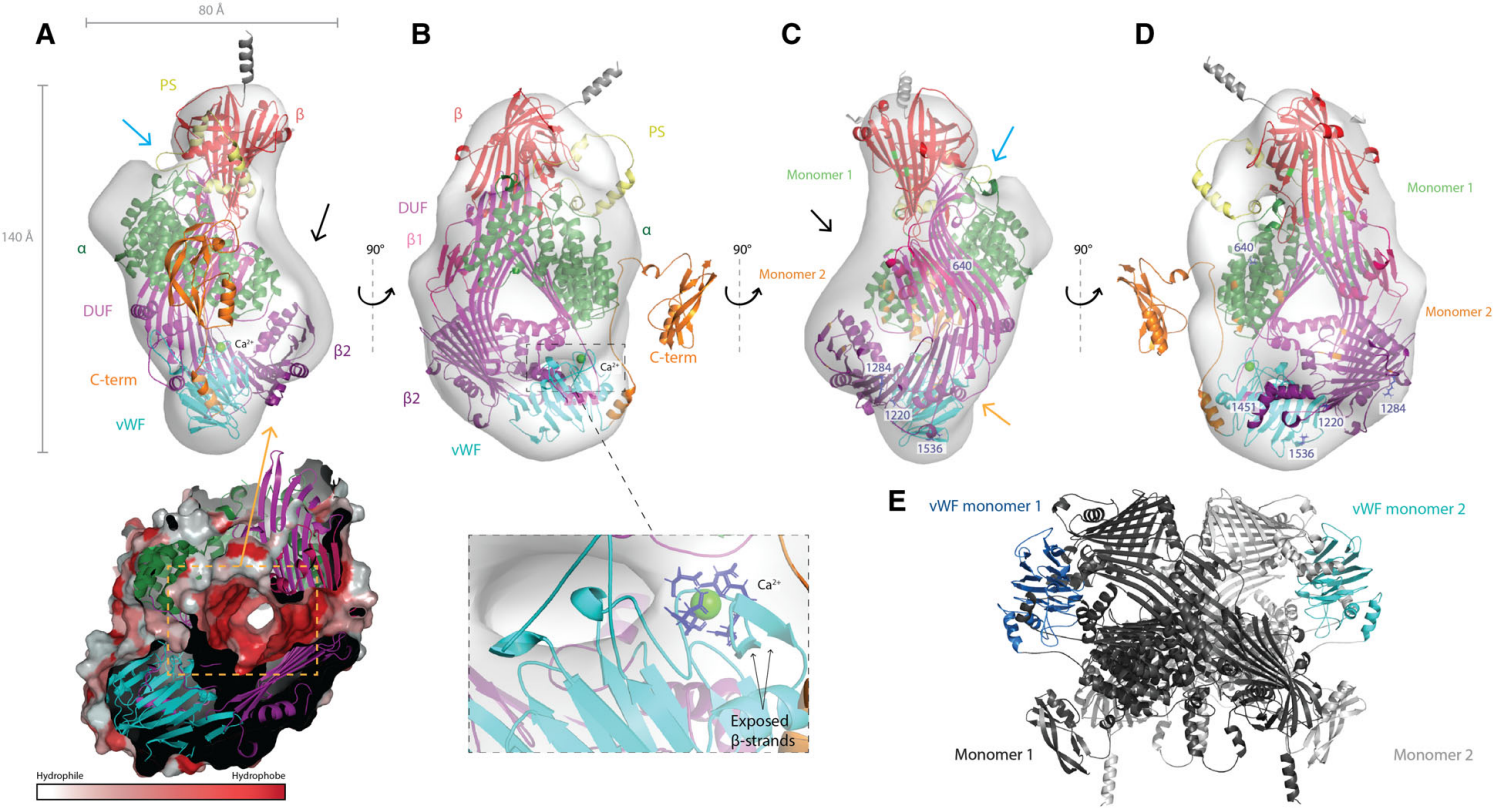
Honey bee vitellogenin final assembly. The EM map is shown as a transparent surface from four different angles and have the AlphaFold model fitted inside. The polyserine linker (PS, yellow), β‐barrel (β, red), α‐helical (α, green), DUF1943 (DUF, magenta), β‐sheet 1 (β1, hot pink), β‐sheet 2 (β2, purple), vWF domain (vWF, cyan), and C‐terminal (C‐term, orange) domains are labeled, as well as the UC (blue arrow), LC (orange arrow), and empty density (black arrow). (A) The measurements of the EM map are shown along the x‐ and y‐axis. The surface of the LC, colored by Eisenberg hydrophobicity scale [44], is shown inside the orange dashed box surrounded by the domains building up the cavity. (B) Here, we zoom in on the Ca^2+^‐binding sites, and show the two exposed β‐strands (black arrows) and their proximity to the LC. (C, D) The five residue positions (640, 1220, 1284, 1451, and 1536) identified as candidates of functional polymorphisms are colored blue and labeled. The conserved residues in honey bee Vg that make polar contacts during dimerization are colored green (monomer 1) and orange (monomer 2). (E) The simulated Vg dimer is shown with monomer 1 (dark gray) and 2 (light gray). The vWF domain is colored in each monomer (monomer 1, dark blue and monomer 2, cyan).

The final model is presented in Fig. 4. The LC serves as the better‐known lipid‐binding site. It is easily accessible, while the hydrophobic core is buried in the EM map (Fig. 4A). The UC is partly built up by the β‐barrel. The vWF domain is placed close to the LC bringing the Ca^2+^‐ion into close proximity to the cavity (Fig. 4B). This is supported by the results produced by the Volume, Area, Dihedral Angle Reporter (VADAR; Fig. S4D). The fractional accessible surface area report shows that the two short β‐strands downstream of the Ca^2+^‐binding site are reported as exposed (r. 145–156 in plot 1, Fig. S4D). The fractional residue volume plot reports a potential cavity in the vicinity of the Ca^2+^‐binding site. In addition to the hydrophobic regions of Vg to be buried in the two cavities, the previously established hydrophilic and positively charged side of the α‐helical domain [3] faces the surface in our model, providing further support for a correct assembly. The polyserine region is also very exposed, favoring the reported dephosphorylation and cleavage events [19]. In the final model, we also mapped out residue positions of interest (Fig. 4C,D). The five functional polymorphisms are in association with a cavity (three in the lipid‐binding site and two in the vWF domain). Anderson et al. [53] specified 12 polar interactions among nine residues on each monomer of lamprey Vg. Seven of these residues are conserved in honey bee Vg, and mapping these to the final model shows them to be accessible to solvent. Simulating the dimerization in pymol with the final model confirms dimerization to be a feasible oligomeric arrangement for honey bee Vg (Fig. 4E). However, re‐fitting the Vg dimer in the EM map results in 33–39% of the atoms inside the contour (Tables S5 and S6). Taken together, this further supports the predicted assembly and demonstrates the EM map to be a representation of monomeric honey bee Vg.

### Vitellogenin oligomerization state

**Fig. 5:**
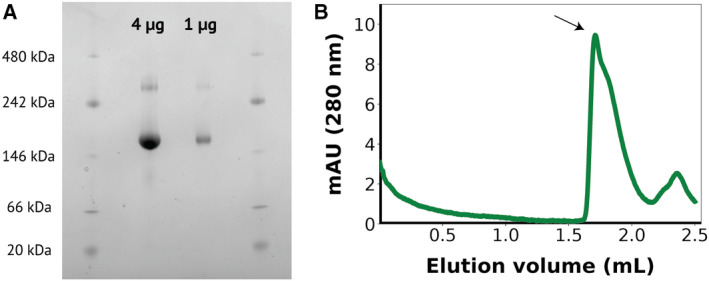
*In vitro* oligomerization state analysis of vitellogenin. (A) BN‐PAGE gel results. Both the bands corresponding to the monomer and the dimer can be observed for Vg loaded in different amounts. (B) SEC elution profile for purified Vg. The peak containing Vg is labeled with an arrow corresponding to an elution volume consistent with monomeric Vg.

While lamprey Vg forms a dimer with a modest 245 Å^2^ hydrophobic interface in the crystal structure [24], mixed evidence exists for the oligomerization status of honey bee Vg. As described above, the negative‐stain EM map with a resolution of 27 Å supports Vg to be monomeric since only one Vg molecule can be placed in the EM map, even at low contouring level. However, the sole known experimentally solved structure suggests that Vg can appear as a dimer [53], at least under some conditions. To further investigate this, we obtained purified Vg from honey bees and evaluated two different amounts using BN‐PAGE (Fig. 5A). The lower molecular weight band (151 kDa) constitutes most of the material in the sample and is assumed to be monomeric Vg. The additional weaker band with higher molecular weight (345 kDa) is assumed to be a minor fraction of dimeric Vg. Contamination by other proteins in the sample seems unlikely since only one band for Vg can be observed from the sample in a denaturing PAGE (not shown). Next, we performed SEC (Fig. 5B), and the content of the concentrated fractions was analyzed with BN‐PAGE (not shown). The main peak obtained corresponded to monomeric Vg, and its apparent molecular weight was estimated to be 178 kDa based on the elution volume. No peak corresponding to the dimeric form was obtained, although when the fraction from the main peak was concentrated, it showed on a native blue PAGE both as a monomer and a dimer in similar proportion to that observed in Fig. 5A. Together, these results suggest that Vg can dimerize at higher protein concentrations *in vitro*.

## Discussion

With this study, we aimed to gain more insight into the structure of honey bee Vg and to attempt a full‐length model of the protein. Our results reveal structural features that have not yet been described for Vg in invertebrates.

First, we presented a detailed structural prediction of the vWF domain. Through homology modeling, we identified a potential class II Ca^2+^‐binding site, which appears to be highly conserved across Vg and vWF‐containing species. The Ca^2+^‐ion coordinates with 4 Asp and 2 Asn residues, through their OD1 or OD2 atoms, respectively, except for D1604, which coordinates through its main chain carbonyl O‐atom. In the human WD3 domain, the residue corresponding to D1604 is I1002 (Fig. S2). The side chain of isoleucine is unable to interact meaningfully with calcium [54]. We speculate that the introduction of a sixth calcium‐coordinating residue, aspartate, creates an additional bond to the Ca^2+^‐ion, increasing the interaction and strengthening the coordination. Identifying a total of six coordinating residues and a loop structure in the binding site enabled us to categorize this as a class II site [48].

We were able to present a full‐length structure prediction of an invertebrate Vg. However, our concern about the remaining domains is that the use of distant homologues with low sequence identity can create predictions influenced by the template used. Studies show that general protein folds are well conserved across great phylogenetic distances despite low conservation of the amino acid sequence [55]. Focusing mainly on the general fold and creating several models by using different query sequence lengths, we increased our confidence in the prediction for each domain. The striking similarity between the AlphaFold prediction and the predicted homology model chains validates our modeling results. In addition, AlphaFold provides a confident domain fold of the C‐terminal region, and predicts folds for loop regions missing in the homology model, enabling us to present a 100% complete structure representation of honey bee Vg, with considerable confidence within each domain. Using PowerFit, ADP_EM and chimera, we were able to present a domain assembly of the full‐length structure prediction. The negative‐stain EM map has a low resolution (27 Å), which increases the margin of error. To limit the number of possible orientations, we fitted the homology models according to size, beginning with the largest. We also fitted the previously predicted domains, the crystal structure of lamprey Vg and the AlphaFold prediction to validate our modeled fold and its placement in the EM map. We evaluated each fit based on the scoring, protruding atoms and overlapping fits of separate domains. We concluded that the AlphaFold PowerFit orientation, with the DUF1943 domain, the two downstream β‐sheets and vWF domain oriented around the LC and the β‐barrel and α‐helical subdomain toward the UC (Fig. 3A1), was the most probable representation for honey bee Vg. The energetics for the full‐length model and the separate domains (e.g., whether polar surfaces or hydrophobic surfaces were exposed to the solvent) are logical, as demonstrated for the lipid binding site (Fig. 4A). The final model does not occupy all available density while the C‐terminal region is outside the contour, which represents about 4.6% of the atoms. The position of this domain is not clear as the Alphafold results indicate a flexibility in the connecting loop. The unassigned density in the low‐resolution EM map above the UC could potentially be where the C‐terminal region is positioned (Fig. 4A,C). Honey bee Vg is also found to be phosphorylated and glycosylated, [25] which is not represented in the protein structure and could explain the excess of density.

Both cavities identified in the EM map are compatible with the assembly, and the LC is identified as the lipid‐binding site, which recognizes lipids, possible fragments of gram‐negative and gram‐positive bacteria [24]. The UC, built up partly by the β‐barrel subdomain, has not been described earlier, and whether the UC has similar recognition potential, to the LC is not known. The *in vitro* mutagenesis experiments performed for the human vWF protein [56] illustrate the importance of the Ca^2+^‐binding site for recognition of factor VIII in a blood‐clotting cascade. A study from 2013 shows fbVg to be membrane associated and speculates the receptor binding site to be in the 150 kDa subunit and not in the β‐barrel domain as previously believed [3]. Insect Vg receptors belong to a subfamily of the low‐density lipoprotein receptor family, and calcium interaction has been shown to be essential for ligand association [57, 58]. Our findings support these results and suggest the vWF domain as the potential Vg receptor binding site. Additionally, the vWF domain has been implicated in having adhesive and lubricant properties [59, 60] as seen for vWF and mucin proteins in humans. The structure of the WD3 domain, used as template here, was recently functionally compared to the MUC2 in humans. Since the two proteins shows high structural similarity, Javitt et al. [61] suggest that WD3 has a similar polymerization function and is essential for macromolecular assemblies in the epithelial mucosa and vasculature. Our study shows that the interchain disulfide bonds, essential for oligomerization in the human vWF [26, 56], are not conserved in honey bees. In addition, residues in the β‐barrel and α‐helical domain are interacting in the Vg dimer, and not the vWF domains (Fig. 4E), thereby ruling out this kind of polymerization activity for the vWF domain in honey bees. However, the Ca^2+^‐binding site, the intrachain disulfide bonds and the β‐sandwich are highly conserved, suggesting a similar function in mucosal immunity, as seen for mucins and vWF proteins in humans.

Insects, which have an open circulation system, have developed an efficient coagulation mechanism that is an essential part of their innate immune system [62]. When exposed to invading microbes, a clotting cascade is initiated, trapping and eventually killing the invaders [63]. The hemolymph clot was recently characterized in a Brazilian whiteknee tarantula, showing the main content to be proteins encompassing vWF‐like domains. Sanggaard et al. [64] results also indicate that the clot functional and structural overlaps with such clots observed in insects. We propose that honey bee Vg can initiate or aid in this clotting mechanism, interacting through the vWF domain, and protect honey bees from pathogens and mechanical damage, like in zebrafish Vg [4]. Our identification of three residue positions exhibiting high genetic differentiation in the LC could be a result of adaption to binding substrates present in specific environments. Our results work well with this theory since we also identified the last two functional polymorphisms close to the LC. This suggests that the vWF domain recognizes environmental factors such as pathogens. Specifically, site 1451 (Fig. 4C,D) is in a small hydrophobic pocket close to the Ca^2+^‐binding site. Our MSA shows conservation of hydrophobicity in this position, which is often seen for binding sites. Based on our collected data, this speculation cannot be confirmed, but could form the basis of new experimental work in which this is explored.

Our results suggest that honey bee Vg is predominantly monomeric *in vitro*. First, only one copy of the Vg model could fit into the low‐resolution EM map. Second, SEC analysis showed only one peak, and this corresponded to monomeric Vg. Third, native gel results also showed a higher tendency toward a monomeric state determined by the much weaker 345 kDa band (presumably a dimer). On the contrary, we demonstrated that the seven residues of each monomer that are creating polar contacts during dimerization in lamprey Vg are conserved in honey bee Vg, making it plausible that Vg dimers can form in honey bees in certain cases. We note that no reducing agent was present in the loading buffer or gel, making it possible that dimers are stabilized by disulfide bonds. Additionally, we cannot rule out that high salt concentration in the SEC prevented the formation of the Vg dimer. Taken together, it is difficult to determine whether dimerization occurs *in vivo* or is an artifact of the *in vitro* conditions, as dimerization occurs frequently in a high concentration sample containing just one type of protein [65]. We speculate that dimerization can be dose‐dependent and thus become more prevalent at elevated Vg concentration. The concentration of Vg in honey bee hemolymph has been reported as high as 100 μg·µL^−1^, illustrating that the protein is highly soluble [66]. More efforts are needed to conclude the oligomeric state of Vg in honey bees and to evaluate earlier evidence describing honey bee Vg to be monomeric [57, 67].

To summarize, our study presents new evidence of the full‐length protein and domain assembly for honey bee Vg. We are thus able to identify properties and describe the structural landscape of the large and versatile protein. Our results verify a second cavity of honey bee Vg in addition to the well described lipid‐binding cavity and describe the structural units potentially forming this cavity. As a result, we are able to suggest the possibility that the vWF domain contributes to the immune system of honey bees, which is currently of global concern due to declining pollinator numbers. Efforts are being made to generate a higher resolution and up‐to‐date EM map, which could be used to preform molecular dynamic flexible fitting and enable studies of Vg protein–protein interactions and ligand binding. Our findings encourage future initiatives in investigating this domain together with the full‐length protein to unravel some of the questions asked here.

## Conflict of interest

The authors declare no conflict of interest.

## Author contributions

VL executed homology modeling, structure predictions, rigid‐body fitting, and purification of honey bee Vg and ØH and GVA supervised the research. EH‐G done the negative staining and generation of the EM map. MM‐C and ESC performed native gel and SEC and HL supervised the research. VL wrote the manuscript with assistance from ØH and GVA. All authors contributed to the manuscript.

## Supporting information


**Fig. S1.** Domain architecture of honey bee and lamprey vitellogenin. The N‐term (green), DUF1943 (pink) and vWF (blue) domains are conserved in both species, as well as the two structural subdomains, β‐barrel (red arrow) and α‐helical domain (dark green curved line). A) Honey bee Vg contains a proteolytic cleavage site, polyserine region (yellow S) linking the two subdomains. The five residue‐positions (640, 1220, 1284, 1451 and 1536) identified to be candidates of functional polymorphisms are marked (brown stars). B) Lamprey Vg contains an addition domain, DUF1943 (purple). The yolk protein organization of IuVg is shown as gray boxes; lipovitellin heavy chain (LvH), Phosvitin (Pv), lipovitellin light chain (LvL), β‐Component (β‐C) and C‐terminal coding region (CT). The dotted lines indicate that these regions (Pv, β‐C and CT) are missing from the crystallographic structure (PDB ID: 1LSH).
**Fig. S2.** Multiple sequence and structural alignment. The coloring for the conserved residues/regions, gaps and secondary structure annotations are explained in the green box. The conserved Ca2+‐binding region are colored in two shades of pink, dark pink is more conserved compared to the lighter pink. A) Extraction of the MSA. The original residue numbering for honey bee Vg is included on top. B) The final structural alignment with the original residue numbering included above each sequence. The annotations are retrieved from the template (PDB ID: 6N29). Both figures are created in Geneious Prime (v. 2019.0.3) and Adobe illustrator (v. 24.0.02).
**Fig. S3.** ProCheck summary, local quality estimate and Ramachandran plots. A) The ProCheck quality evaluations summarized and categorized by calculation results. The ideal residue values and standard deviation for any given model are derived from Morris et al. 1992.^1^ The max deviation, in residues properties, is calculated from the mean value of the residue‐by‐residue listing values (Fig. S4E) of the full‐length structure. The number of bad contacts is defined as the non‐bonded atoms at a distance of <= 2.6 Å. The bond length and angles are calculated in similar manner as the max deviation, but the ideal values are based on Engh and Huber 1991.^2^ The Morris et al. (1992) class summarizes the three above stereochemical parameters by assigning a number between 1 (best) to 4 (worst), indicating the overall quality of the model. B) Local QMEAN results are presented. The first plot is analysis of the template (green), while the second is analysis of the target modeled interactively (cyan) and automatically (red). The Ca^2+^‐binding region (magenta Ca), the Cys residues forming the intra‐chain disulfide bridges (orange, C) are in the higher quality region, while Ω5‐7 (black) are in the lower quality region. The local score is calculated for each residue in the model and the average local score for the template is 0.93 ± 0.07, while the target average score is 0.40 ± 0.07 (cyan) and 0.44 ± 0.06 (red). C) The Ramachandran plot produced by ProCheck. The plot on the left is the template (PDB ID: 6N29), while the target (honey bee vWF domain) is on the right. Below each plot, the statistic is presented.
**Fig. S4.** Global quality estimate, VADAR plots and ProCheck residue listing. A‐C) The plots of the global QMEAN have the QMEAN4 scores for a set PDB structures plotted (gray dots) with the QMEAN4 score along the x‐axis and the number of residues in the structures as long the y‐axis. The global scores value QMEAN4 range from 0 to 1, where 1 is good. A) Analysis of the template (red star) and the QMEAN4 value is written on the plot. B) Analysis of the interactively homology modeled (red star) structure and C) The automatically homology modeled (red star) structure from MODELLER. D) Four different analyses were performed by VADAR, presented in one plot each, with the template (gray) compared to the target (green). Plot 1: a low fractional ASA score indicates a buried residue, while a score above 0.5 (dotted black line) indicates an exposed residue. A score above 1.0 (red line) indicates a problem in the structure. Plot 2: When a protein structure is efficiently packed the score should be around 1.0 ± 0.1. A score above 1.2 (blue line) or below 0.8 (red line) could indicate a poor refinement or identify cavities. Plot 3: Each residue is assigned a score between 0‐3 (high is good quality) for three different measurements (torsion angle, omega angle and fractional volume). The total quality score for each residue can be from 0‐9 and the threshold for a good quality is set to 6 (red line). Plot 4: Calculates the 3D quality of each residue based on its environment and gives a score between 0‐9 (high is good quality), and the threshold for a good quality is set to 4 (red line). E) The Residue‐by‐Residue listing for ProCheck lists all residues in a structure and present all calculations for each. A short example is shown here for the first six residues in the target structure. Each value is compared to the ideal values which is noted on top. The deviating values are marked with * (one standard deviation) and + (half a standard deviation) sign. For example, the omega dihedral angle of residue S1443 is 16.9 standard deviation away from the ideal value, which is a result from the loop building of Ω1.
**Fig. S5.** Comparison of vWF homology models. A) The sort region around the Ca^2+^‐binding site (Ca^2+^‐ion, green) is shown from the interactively modeled (cyan) structure and the automatically modeled (gray) structure. The Cys‐residues (C1444, C1466, C1598 and C1634) and Ca^2+^‐binding residues are shown as yellow/cyan (interactively) and orange/magenta (automatically) sticks. The missing C1634 and β‐strands in the automatically modeled structure are shown (gray arrows). B) All the Ca^2+^‐binding residues are in the same orientation in both models (light blue: interactively and light pink: automatically), except N1607. The interactions to the Ca^2+^‐ion is shown as yellow dotted lines and measured (Å) for N1607.
**Fig. S6.** Comparison of homology models from MODELLER and RaptorX. A) The N‐terminal domain: Model 1 (green) aligned with Model 4 (red), 5 (yellow) and 6 (forest green). B) The DUF1943 domain: Model 1 (magenta) aligned with Model 8 (cyan), Model 7 (orange) and Model 9 (blue). The identified curve in the longer β‐sheet in Model 1, 8 and 9 and the missing curve in Model 7 is marked with arrows. C) The DUF1943 domain Model 1 (magenta), the downstream region residue 1060 to 1140 of Model 9 (hot pink) and the loop region (gray). D) The undetermined domain: Model 2 (purple) aligned with Model 9 (blue), with the long loop region (gray). E) The interactively homology model of vWF domain (cyan) with the C‐terminal region from Model 9 (orange).
**Fig. S7.** RaptorX structural prediction of full‐length honey bee vitellogenin. A) The β‐barrel subdomain (red), the polyserine linker (yellow), the α‐helical subdomain (forest green), the DUF1943 domain (magenta), elongation of the DUF1943 domain (hot pink arrow), the undetermined structural region (purple), the vWF domain (cyan) and the C‐terminal region (orange) are generated as one full‐length model. The two loop regions (gray arrows) are also predicted. B) Domain 1 to 6 from Table S7 are colored red, cyan, purple, blue, green and orange, respectively, and if templates was used, the PDB ID is written in parenthesis.
**Fig. S8.** AlphaFold output. A) The number of sequence hits in the MSA produced by AlphaFold, is plotted per residue. The average number of hits per residue (gray dotted line), and the threshold at 100 sequence per residue (red dotted line) is marked. B) The plDDT score for the five outputted models by AlphaFold is plotted per residue, and the average plDDT score per model is listed to the right, which produces the rank from 0 (best) to 4 (worst). C) The ranked models are aligned, colored by the same coloring scheme in panel B, and the consistently folded domains (β‐barrel (β), α‐helical (α), DUF1943 (DUF), β‐sheet 1 (β1), β‐sheet 2 (β2) and vWF domain (vWF)) are labeled in bold letters, while the more variable domains (N‐terminal, polyserine linker (PS) and C‐terminal) are labeled in grey letters. D) The final homology model domains (β‐barrel (red), polyserine linker (yellow), α‐helical (green), DUF1943 (magenta), β‐sheet 1 (hotpink), β‐sheet 2 (purple), vWF (cyan, Ca^2+^‐ion shown as green sphere) and C‐terminal domain (orange) is aligned to their respective domains in the top ranked AlphaFold prediction (grey). The grey brackets to the lower right indicate the region where AlphaFold have predicted a fold for the main missing atoms in the homology model.
**Fig. S9.** EM map validation. A) Map visualization to allow visual inspection of the internal detail of the map and identification of artifacts. The primary map, central slices of the map and largest variance of the map is shown in three orthogonal directions. The 3D surface view of the primary map at recommended contour level 0.07. B) Statistical analysis of the map. In the first graph the map‐value distributions is plotted in 128 intervals along the x‐axis, and the y‐axis is logarithmic. The spike around 0 indicate that the volume has been masked. The second graph shows how the enclosed volume varies with the contour level. The volume at the recommended contour (red line) is 289 nm3; this corresponds to an approximate mass of 261 kDa. C) The provided Fourier‐Shell Correlation (blue) is plotted together with the reported resolution, (black line, *Reported resolution corresponds to spatial frequency of 0.037 Å^‐1^). A curve is displayed for the half‐bit criterion (dashed red), in addition to lines showing the 0.143 gold standard cut‐off (dashed orange line) and 0.5 cut‐off (green dotted line). All the graphs are assembled from the EmDataBank map validation report (copy included).
**Fig. S10.** Rigid‐body fitting for honey bee vitellogenin homology models. A) The EM map is shown as a gray surface. The distinct cavity creases are marked with stars and arrows, upper cavity (blue) and lower cavity (yellow). The four curves in the surface are labeled (top, base, left and right). B) The correlation score and precent of atoms outside the contour calculated by Chimera was plotted for each fit from PowerFit (PF, blue) and ADP_EM (ADP, pink), and ranked according to the correlation score (dark color: highest score, light color: second highest score). C‐E) The fits from the full‐length homology model, RaptorX and chain A is presented inside the EM map, with the same coloring scheme as in panel B. The β‐barrel (β), α‐helical (α), DUF1943 (DUF), vWF and C‐terminal (C‐t) domains are labeled. If the domain is outside of the contour it is noted by a “*”‐mark. F) The fits of chain B to E separately with the same coloring scheme as in panel B, but they are labeled according to chains and not domains.
**Fig. S11.** Rigid‐body fitting of chain A to C and D. A) The correlation score and precent of atoms outside the contour calculated by Chimera was plotted for each fit from PowerFit (PF, blue) and ADP_EM (ADP, pink), and ranked according to the correlation score (dark color: highest score, light color: second highest score). B) The EM map are shown as a transparent surface, and the fits of chain A to C from PF and ADP are shown as cartoons and colored by method and scores (dark blue: PF1, light blue: PF2, dark pink: ADP1, light pink: ADP2). The β‐barrel (β), α‐helical (α) and DUF1943 (DUF) domains are labeled. C) The EM map and the fits of chain D is shown in same coloring scheme as in panel B. The label is marked with “*” if the fit is outside the contour of the EM map. D) The EM map are shown as a surface, less transparent than in panel B, with the fits of chain A to C (1: PF2 and ADP1, 2: PF1 and ADP2) in the same coloring scheme as in panel B. The EM map is shown at four different angels, and arrows points to secondary structure elements from β, α or DUF domain which are outside the contour of the EM map.
**Fig. S12.** Rigid‐body fitting for previously published homology models and a distant homologue. A) The same plot as in Fig. S10 for the β‐barrel and α‐helical subdomains, and the crystal structure of lamprey Vg (1LSH). B‐D) Same presentation and coloring scheme as in Fig. S10C‐S10F.
**Table S1.** Alignment parameters.
**Table S2.** List of species used in the multiple sequence alignment.
**Table S3.** Loop building based on gaps in the structural alignment.
**Table S4.** Edited residues during quality control.
**Table S5.** Rigid‐body fitting scores from PowerFit and Chimera.
**Table S6.** Rigid‐body fitting scores from ADP_EM and Chimera.
**Table S7.** RaptorX structure prediction.

**Appendix S1.** wwPDB EM Validation Summary Report.

**Appendix S2.** Top ranked Vitellogenin model by AlphaFold.

## Data Availability

The data that support the findings of this study are available in the supplementary material of this article (Tables S1–S7, Figs S1–S12, and the EM map validation report [Appendix S1]). The structural data from homology modeling of the vWF domain are openly available at ModelArchive https://modelarchive.org/doi/10.5452/ma‐sfueo (access code: okHs98Pcl2), and the structural data from AlphaFold are available in the supplementary material of this article.
