# Supplementary material for: Structure prediction of honey bee vitellogenin: a multi‐domain protein important for insect immunity
Source: FEBS Open Bio. 2021 Oct 31;12(1):51–70. doi: 10.1002/2211-5463.13316 (PMC8727950; doi:10.1002/2211-5463.13316)
Supplement: Supplementary file 1 — Fig. S1. Domain architecture of honey bee and lamprey vitellogenin. The N‐term (green), DUF1943 (pink) and vWF (blue) domains are conserved in both species, as well as the two structural subdomains, β‐barrel (red arrow) and α‐helical domain (dark green curved line). A) Honey bee Vg contains a proteolytic cleavage site, polyserine region (yellow S) linking the two subdomains. The five residue‐positions (640, 1220, 1284, 1451 and 1536) identified to be candidates of functional polymorphisms are marked (brown stars). B) Lamprey Vg contains an addition domain, DUF1943 (purple). The yolk protein organization of IuVg is shown as gray boxes; lipovitellin heavy chain (LvH), Phosvitin (Pv), lipovitellin light chain (LvL), β‐Component (β‐C) and C‐terminal coding region (CT). The dotted lines indicate that these regions (Pv, β‐C and CT) are missing from the crystallographic structure (PDB ID: 1LSH). Fig. S2. Multiple sequence and structural alignment. The coloring for the conserved residues/regions, gaps and secondary structure annotations are explained in the green box. The conserved Ca2+‐binding region are colored in two shades of pink, dark pink is more conserved compared to the lighter pink. A) Extraction of the MSA. The original residue numbering for honey bee Vg is included on top. B) The final structural alignment with the original residue numbering included above each sequence. The annotations are retrieved from the template (PDB ID: 6N29). Both figures are created in Geneious Prime (v. 2019.0.3) and Adobe illustrator (v. 24.0.02). Fig. S3. ProCheck summary, local quality estimate and Ramachandran plots. A) The ProCheck quality evaluations summarized and categorized by calculation results. The ideal residue values and standard deviation for any given model are derived from Morris et al. 1992.1 The max deviation, in residues properties, is calculated from the mean value of the residue‐by‐residue listing values (Fig. S4E) of the full‐length structure. The number of bad c [file FEB4-12-51-s002.docx]

## Supplementary material

### Table S1. Alignment parameters

The pairwise alignment was performed using EMBOSS Needle with default settings. The multiple alignment was performed using BLAST with default settings. The structural alignment was performed and altered in spdbv. The sequence identify is higher (30.6 %) in spdbv due to different default penalty scores.

| Alignment | Matrix | Gap Open | Gap extend | End Gap penalty | End Gap open | End gap Extend |
| --- | --- | --- | --- | --- | --- | --- |
| Emboss | BLOSUM62 | 10 | 0.5 | false | 10 | 0.5 |
| BLAST | Automatically selected | 11 | 1 | N/A | N/A | N/A |
| Spdbv | PAM200 | 6 | 4 | N/A | N/A | N/A |

### Table S2. List of species used in the multiple sequence alignment

| UniProt ID | Species |
| --- | --- |
| sp\|Q868N5.1\|VIT_APIME | *Apis mellifera* |
| sp\|Q2VQM6.1\|VIT2_SOLIN | *Solenopsis invicta* |
| sp\|Q7Z1M0.1\|VIT1_SOLIN | *Solenopsis invicta* |
| sp\|Q2VQM5.1\|VIT3_SOLIN | *Solenopsis invicta* |
| sp\|Q9U8M0.1\|VIT1_PERAM | *Periplaneta americana* |
| sp\|Q9BPS0.1\|VIT2_PERAM | *Periplaneta americana* |
| sp\|Q16927.2\|VIT1_AEDAE | *Aedes aegypti* |
| sp\|Q05808.1\|VIT_ANTGR | *Anthonomus grandis* |
| sp\|Q27309.1\|VIT_BOMMO | *Bombyx mori* |
| sp\|P55155.2\|VIT1_CAEEL | *Caenorhabditis elegans* |
| sp\|P05690.5\|VIT2_CAEEL | *Caenorhabditis elegans* |
| sp\|P80012.2\|VWF_BOVIN | *Bos taurus* |
| sp\|Q28833.2\|VWF_PIG | *Sus scrofa* |
| sp\|P04275\|VWF_HUMAN | *Homo sapiens* |
| sp\|Q8CIZ8\|VWF_MOUSE | *Mus musculus* |
| sp\|Q28295.2\|VWF_CANLF | *Canis lupus* |

### Table S3. Loop building based on gaps in the structural alignment

The table shows the loop building preformed in spdbv. The gaps are numbered according to the structural alignment, and the specific residues, how many and type of gap is noted. The last three columns list the parameters given by spdbv for the selected loop (except gap 5-7, were loop building was unsuccessful).

| Gap | Target vWF | Number of residues | Type | *Ab initio* Loop | | |
| --- | --- | --- | --- | --- | --- | --- |
|  |  |  |  | Clash Score | Pair potential | Force field energy |
| 1 | S1443 | 1 | Insertion | 4 | -3.15 | 1563.8 |
| 2 | D1447-K1448 | 1 | Deletion | -3 | -2.27 | 117.3 |
| 3 | P1460 | 1 | Insertion | -6 | -1.90 | 674.8 |
| 4 | H1482-N1483 | 5 | Deletion | -4 | 2.80 | 2138.5 |
| 5 | V1494-G1504 | 11 | Insertion | Removed res. 1494-1504 from sequence | | |
| 6 | I1517-Y1526 | 10 | Insertion | Removed res. 1515-1522 from sequence | | |
| 7 | V1537-Y1544 | 8 | Insertion | Removed res. 1537-1541 from sequence | | |
| 8 | D1561 | 1 | Insertion | -8 | -0.81 | -49.6 |
| 9 | K1570-F1571 | 1 | Deletion | -3 | 0.26 | 102.1 |
| 10 | L1575-A1576 | 2 | Insertion | -6 | 0.53 | 16872.0 |
| 11 | D1589-Y1590 | 1 | Deletion | -7 | 0.64 | 30.3 |
| 12 | I1630 | 1 | Insertion | -8 | 0.33 | 46059.2 |

### Table S4. Edited residues during quality control

Based on the Ramachandran plot and bad contacts detected by ProCheck, the listed residue rotamer option were edited to the most optimal rotamer. Regions in the Ramachandran Plot can be defined as: A - Core alpha, a - Allowed alpha, ~a - Generous alpha, B - Core beta, b - Allowed beta, ~b - Generous beta, L - Core left-handed alpha, l - Allowed left-handed alpha, ~l - Generous left-handed alpha, p - Allowed epsilon, ~p - Generous epsilon, XX - Outside major areas.

| Detected by Ramachandran Plot | Detected region | Region edited to | Detected by ProCheck “Bad Contacts” Edited to a more optimal rotamer option to avoid clashes |
| --- | --- | --- | --- |
| R1450 | XX | p | K1448 |
| H1482 | ~b | ~a | K1457 |
| E1484 | XX | b | Y1459 |
| K1485 | XX | L | L1463 |
| L1486 | ~l | ~l | M1471 |
| Q1526 | XX | ~a | N1478 |
| F1563 | XX | A | I1477 |
| K1570 | XX | ~a | E1507 |
| F1571 | XX | ~a | T1524 |
| L1577 | XX | ~b | V1529 |
| D1578 | XX | l | F1531 |
| D1589 | XX | ~b | I1536 |
| S1631 | ~l | ~l | V1547 |
| S1632 | ~l | ~l | D1572 |
|  | | | L1575 |
|  |  |  | M1583 |
|  |  |  | Y1592 |
|  |  |  | V1610 |
|  |  |  | Y1627 |

### Table S5. Rigid-body fitting scores from PowerFit and Chimera.

The fits presented generated in PowerFit are ranked (Fit), which is also used when fitted the models in Chimera. Scores from PowerFit presented is the Cross Correlation score (CSS), Fisher z-score (Fish-z), the z-score as factor of standard deviations (rel-z) and the sigma difference to the best fit ((z_1_-z_N_)/σ). Chimera also generates Correlation score (C), in addition to the average map value (AVM). A count of the number of atoms outside the contour, from the total atoms in the model is generated. The percentage of atoms outside is also included.

|  | PowerFit | | | | | Chimera fit-in-map | | | | |
| --- | --- | --- | --- | --- | --- | --- | --- | --- | --- | --- |
| Model | Fit | CCS | Fish-z | rel-z (z/σ) | (z_1_-z_N_)/σ | C | AVM | Outside | Total | % |
| Full-length Vg | 1  2  3 | 0.415  0.391  0.389 | 0.442  0.413  0.411 | 32.2  30.0  29.9 | 0.00  2.14  2.26 | 0.7575  0.7417  0.8114 | 0.06003  0.05491  0.06224 | 6250  6984  6015 | 13277  13277  13277 | 47  53  45 |
| RaptorX | 1  2  3 | 0.371  0.356  0.352 | 0.390  0.372  0.368 | 28.2  26.9  26.6 | 0.00  1.28  1.58 | 0.6961  0.7265  0.6971 | 0.04817  0.05336  0.05010 | 8882  7981  8135 | 14381  14381  14381 | 62  55  57 |
| Chain A | 1  2  3 | 0.460  0.458  0.448 | 0.498  0.495  0.482 | 28.0  27.9  27.1 | 0.00  0.15  0.89 | 0.8877  0.8465  0.8685 | 0.08099  0.07797  0.08124 | 2064  2558  1902 | 8301  8301  8301 | 25  31  23 |
| Chain B | 1  2  3 | 0.758  0.700  0.696 | 0.992  0.867  0.859 | 14.9  13.0  12.9 | 0.00  1.87  1.99 | 0.7051  0.6035  0.7116 | 0.01949  0.01896  0.01910 | 662  662  662 | 662  662  662 | 100  100  100 |
| Chain C | 1  2  3 | 0.602  0.598  0.558 | 0.696  0.690  0.629 | 18.3  18.1  16.6 | 0.00  0.16  1.74 | 0.8163  0.8167  0.7800 | 0.09602  0.09401  0.09085 | 32  62  344 | 1714  1714  1714 | 2  4  20 |
| Chain D | 1  2  3 | 0.695  0.690  0.677 | 0.858  0.848  0.824 | 20.2  20.0  19.4 | 0.00  0.24  0.81 | 0.1898  0.9022  0.4387 | 0.00622  0.09620  0.00542 | 1376  311  1376 | 1376  1376  1376 | 100  23  100 |
| Chain E | 1  2  3 | 0.538  0.536  0.527 | 0.602  0.598  0.586 | 12.1  12.1  11.8 | 0.00  0.07  0.32 | 0.8309  0.6326  0.6617 | 0.09487  0.01540  0.01798 | 138  1224  1224 | 1224  1224  1224 | 11  100  100 |
| Chain A to C | 1  2  3 | 0.449  0.433  0.428 | 0.483  0.464  0.457 | 32.0  30.7  30.3 | 0.00  1.29  1.70 | 0.8776  0.8778  0.8474 | 0.07667  0.07665  0.07520 | 3362  3362  3624 | 10677  10677  10677 | 31  31  34 |
| β-barrel | 1  2 | 0.619  0.602 | 0.723  0.696 | 23.4  22.5 | 0.00  0.88 | 0.8235  0.8137 | 0.09408  0.09315 | 166  208 | 4824  4824 | 3  4 |
| α-helical | 1  2 | 0.667  0.666 | 0.806  0.803 | 25.8  25.7 | 0.00  0.09 | 0.8277  0.8277 | 0.0927  0.0951 | 252  68 | 3678  3678 | 7  2 |
| 1LSH | 1  2 | 0.466  0.461 | 0.505  0.499 | 33.7  33.3 | 0.00  0.42 | 0.8987  0.8820 | 0.08254  0.07918 | 2688  3185 | 10935  10935 | 25  29 |
| AlphaFold | 1  2  3 | 0.451  0.427  0.423 | 0.485  0.457  0.452 | 39.7  37.3  36.9 | 0.00  2.34  2.76 | 0.8552  0.8767  0.8747 | 0.07000  0.07414  0.07413 | 10724  8834  8821 | 28204  28204  28204 | 38  31  31 |
| Vg dimer  AlphaFold | 1  2  3 | 0.326  0.324  0.310 | 0.338  0.336  0.321 | 38.8  38.5  36.8 | 0.00  0.37  2.03 | 0.7386  0.6609  0.6989 | 0.04643  0.04007  0.04340 | 35252  37296  36184 | 56408  56408  56408 | 62  66  64 |

### Table S6. Rigid-body fitting scores from ADP_EM and Chimera.

The fits generated in ADP_EM are presented as in Table S5. The fits are ranked (Fit) and given a correlation score (C). The Chimera Fit-in-map scores are presented as in Table S5.

|  | ADP_EM | | Chimera fit-in-map | | | | |
| --- | --- | --- | --- | --- | --- | --- | --- |
| Model | Fit | C | C | AVM | Outside | Total | % |
| Full-length Vg | 1  2  3 | 0.626  0.615  0.595 | 0.8003  0.8101  0.7877 | 0.06121  0.06224  0.06366 | 6025  6005  6195 | 13264  13231  12545 | 45  45  49 |
| RaptorX | 1  2  3 | \| 0.623 \| \| --- \| \| 0.622 \| \| 0.603 \| | \| 0.7126 \| \| --- \| \| 0.6864 \| \| 0.7153 \| | \| 0.05419 \| \| --- \| \| 0.05103 \| \| 0.05426 \| | \| 7800 \| \| --- \| \| 7846 \| \| 7838 \| | \| 13958 \| \| --- \| \| 13768 \| \| 13916 \| | 56  57  56 |
| Chain A | 1  2  3 | \| 0.624 \| \| --- \| \| 0.522 \| \| 0.474 \| | \| 0.8657 \| \| --- \| \| 0.8438 \| \| 0.8842 \| | \| 0.08148 \| \| --- \| \| 0.07940 \| \| 0.08103 \| | \| 2062 \| \| --- \| \| 2479 \| \| 2032 \| | \| 8301 \| \| --- \| \| 8301 \| \| 8301 \| | \| 25 \| \| --- \| \| 30 \| \| 24 \| |
| Chain B | 1  2  3 | \| 0.700 \| \| --- \| \| 0.559 \| \| 0.529 \| | \| 0.7851 \| \| --- \| \| 0.7892 \| \| 0.7813 \| | \| 0.09304 \| \| --- \| \| 0.09314 \| \| 0.10300 \| | \| 139 \| \| --- \| \| 135 \| \| 0 \| | \| 662 \| \| --- \| \| 662 \| \| 662 \| | \| 21 \| \| --- \| \| 20 \| \| 0 \| |
| Chain C | 1  2  3 | \| 0.573 \| \| --- \| \| 0.558 \| \| 0.522 \| | \| 0.8190 \| \| --- \| \| 0.8186 \| \| 0.8140 \| | \| 0.09597 \| \| --- \| \| 0.09603 \| \| 0.09557 \| | \| 33 \| \| --- \| \| 8 \| \| 25 \| | \| 1714 \| \| --- \| \| 1714 \| \| 1714 \| | \| 2 \| \| --- \| \| 0 \| \| 1 \| |
| Chain D | 1  2  3 | \| 0.681 \| \| --- \| \| 0.498 \| \| 0.489 \| | \| 0.9204 \| \| --- \| \| 0.9189 \| \| 0.8255 \| | \| 0.09540 \| \| --- \| \| 0.09542 \| \| 0.10040 \| | \| 132 \| \| --- \| \| 132 \| \| 5 \| | \| 1376 \| \| --- \| \| 1376 \| \| 1376 \| | \| 10 \| \| --- \| \| 10 \| \| 0 \| |
| Chain E | 1  2  3 | \| 0.694 \| \| --- \| \| 0.533 \| \| 0.517 \| | \| 0.7703 \| \| --- \| \| 0.8118 \| \| 0.8030 \| | \| 0.06953 \| \| --- \| \| 0.09954 \| \| 0.09483 \| | \| 495 \| \| --- \| \| 32 \| \| 55 \| | \| 1224 \| \| --- \| \| 1224 \| \| 1224 \| | \| 40 \| \| --- \| \| 3 \| \| 4 \| |
| Chain A to C | 1  2  3 | \| 0.64 \| \| --- \| \| 0.628 \| \| 0.578 \| | \| 0.8536 \| \| --- \| \| 0.8776 \| \| 0.8503 \| | \| 0.07537 \| \| --- \| \| 0.07666 \| \| 0.07322 \| | \| 3734 \| \| --- \| \| 3353 \| \| 3751 \| | \| 10677 \| \| --- \| \| 10677 \| \| 10677 \| | \| 35 \| \| --- \| \| 31 \| \| 35 \| |
| β-barrel | 1  2 | \| 0.621 \| \| --- \| \| 0.537 \| | \| 0.8179 \| \| --- \| \| 0.8323 \| | \| 0.08273 \| \| --- \| \| 0.96010 \| | \| 1410 \| \| --- \| \| 75 \| | \| 4824 \| \| --- \| \| 4824 \| | \| 29 \| \| --- \| \| 2 \| |
| α-helical | 1  2 | \| 0.616 \| \| --- \| \| 0.581 \| | \| 0.8177 \| \| --- \| \| 0.8318 \| | \| 0.09211 \| \| --- \| \| 0.09778 \| | \| 109 \| \| --- \| \| 111 \| | \| 3678 \| \| --- \| \| 3678 \| | \| 3 \| \| --- \| \| 3 \| |
| 1LSH | 1  2 | \| 0.638 \| \| --- \| \| 0.567 \| | \| 0.8984 \| \| --- \| \| 0.8916 \| | \| 0.08254 \| \| --- \| \| 0.08058 \| | \| 2693 \| \| --- \| \| 3057 \| | \| 10935 \| \| --- \| \| 10935 \| | \| 25 \| \| --- \| \| 28 \| |
| AlphaFold | 1  2  3 | 0.640  0.603  0.589 | 0.8603  0.8525  0.8283 | 0.06998  0.07730  0.06922 | 7168  8768  8504 | 24015  24015  24015 | 30  37  35 |
| Vg dimer  AlphaFold | 1  2  3 | \| 0.620 \| \| --- \| \| 0.612 \| \| 0.595 \| | \| 0.6330 \| \| --- \| \| 0.732 \| \| 0.705 \| | \| 0.03619 \| \| --- \| \| 0.04550 \| \| 0.04344 \| | \| 37636 \| \| --- \| \| 34199 \| \| 35558 \| | \| 56408 \| \| --- \| \| 56408 \| \| 56408 \| | \| 67 \| \| --- \| \| 61 \| \| 63 \| |

### Table S7. RaptorX structure prediction.

The resulting full-length structure prediction was compiled of six domains, which in again was based on homology modeling or template-free modeling. The amino acid sequence of honey bee Vg used in each domain are listed, as well as the alignment score, P-value, sequence identity and the PDB IDs of the templates.

| Domain | Vg sequence | Model | Score* | P-value | Seq. ident. | Template |
| --- | --- | --- | --- | --- | --- | --- |
| 1 | 1-1142 | 1/1 | 801 | 1.5e-16 | 12 | 1LSH_A |
| 2 | 1440-1643 | 1/1 | 133 | 1.9e-08 | 21 | 6RBF_A |
| 3 | 1202-1439 | 1/2  2/2 | 141  92 | 1.6e-08  1.0e-05 | 8  6 | 1LSH_B  3WJB_A |
| 4 | 1143-1202 | 1/5  2/5  3/5  4/5  5/5 | 3  3  3  3  3 | N/A  N/A  N/A  N/A  N/A | 0  0  0  0  0 | Template-free  Template-free  Template-free  Template-free  Template-free |
| 5 | 1712-1770 | 1/5  2/5  3/5  4/5  5/5 | 54  54  53  52  52 | 1.2e-03  1.3e-03  1.5e-03  1.7e-03  1.7e-03 | 5  9  10  14  7 | 4YU8_A  4JPH_A  5BPU_A  4NT5_A  2KD3_A |
| 6 | 1644-1712 | 1/5  2/5  3/5  4/5  5/5 | 5  5  5  5  5 | N/A  N/A  N/A  N/A  N/A | 0  0  0  0  0 | Template-free  Template-free  Template-free  Template-free  Template-free |

*Score: The alignment score which can go from 0 to the length of the domain sequence, with 0 indicating the lowest score.

### Supplementary Figures

**
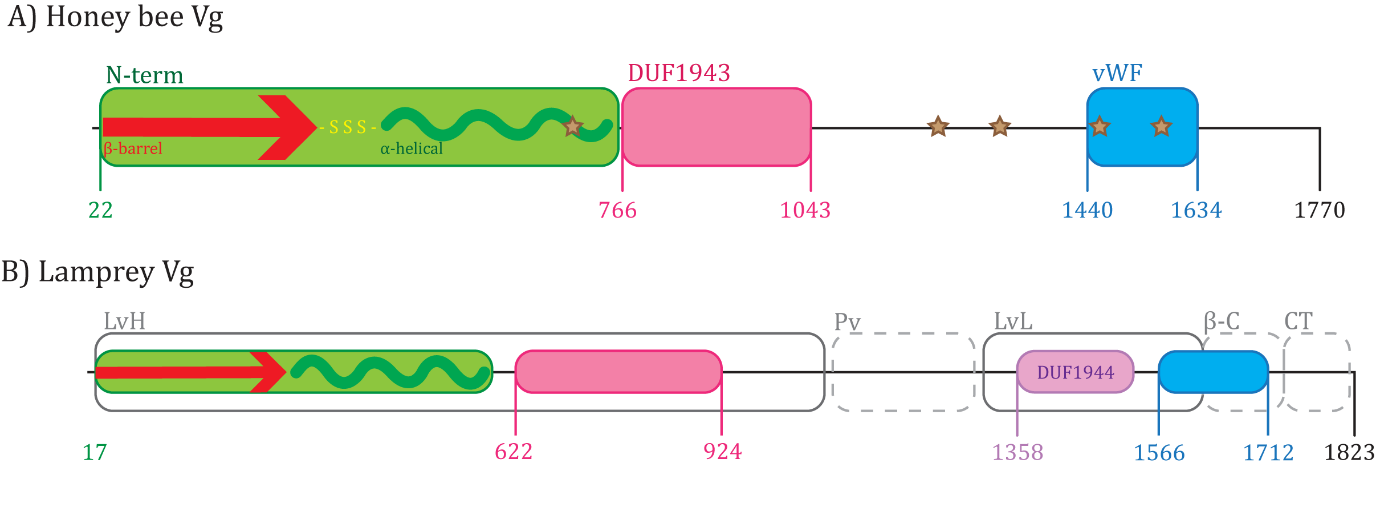
Figure S1. Domain architecture of honey bee and lamprey vitellogenin**. The N-term (green), DUF1943 (pink) and vWF (blue) domains are conserved in both species, as well as the two structural subdomains, β-barrel (red arrow) and α-helical domain (dark green curved line). **A)** Honey bee Vg contains a proteolytic cleavage site, polyserine region (yellow S) linking the two subdomains. The five residue-positions (640, 1220, 1284, 1451 and 1536) identified to be candidates of functional polymorphisms are marked (brown stars). **B)** Lamprey Vg contains an addition domain, DUF1943 (purple). The yolk protein organization of IuVg is shown as gray boxes; lipovitellin heavy chain (LvH), Phosvitin (Pv), lipovitellin light chain (LvL), β-Component (β-C) and C-terminal coding region (CT). The dotted lines indicate that these regions (Pv, β-C and CT) are missing from the crystallographic structure (PDB ID: 1LSH).

**
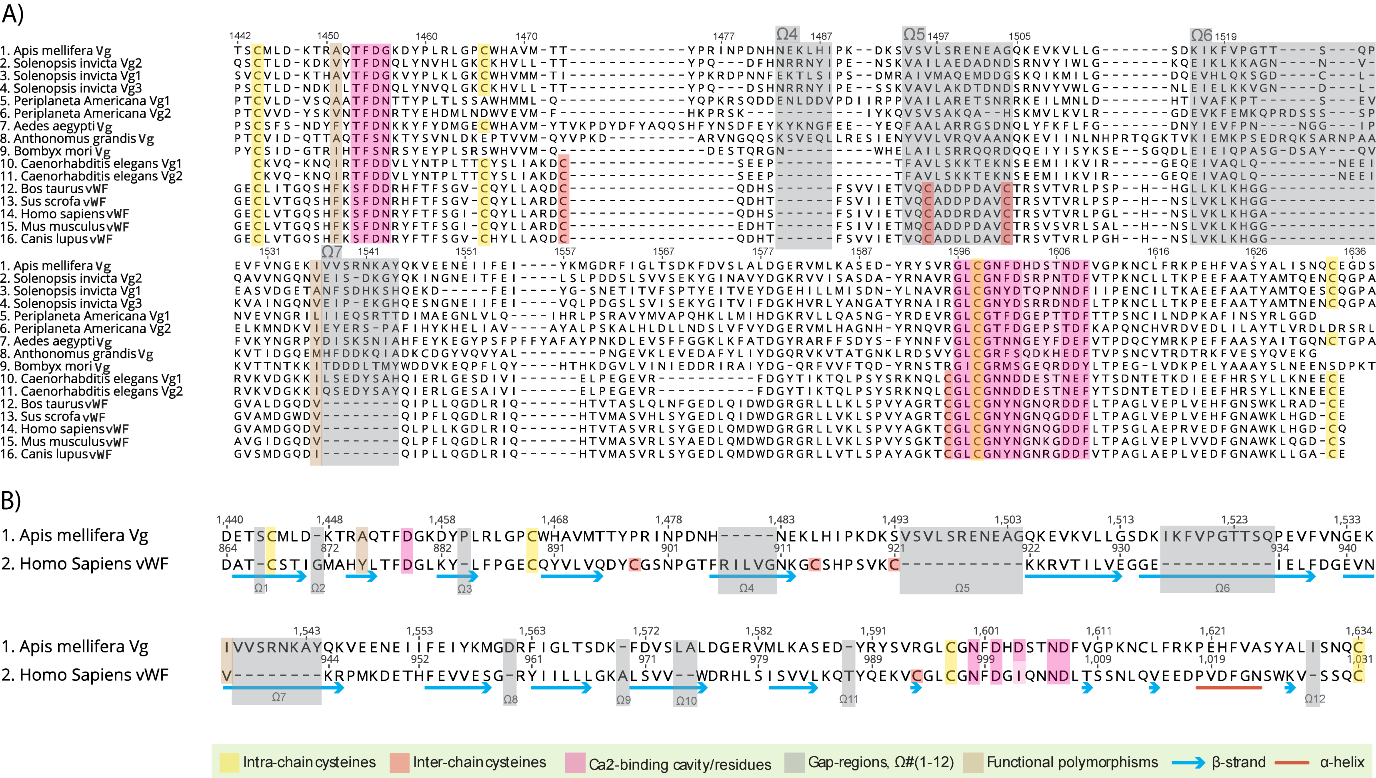
**

**Figure S2. Multiple sequence and structural alignment.** The coloring for the conserved residues/regions, gaps and secondary structure annotations are explained in the green box. The conserved Ca2+-binding region are colored in two shades of pink, dark pink is more conserved compared to the lighter pink. **A)** Extraction of the MSA. The original residue numbering for honey bee Vg is included on top. **B)** The final structural alignment with the original residue numbering included above each sequence. The annotations are retrieved from the template (PDB ID: 6N29). Both figures are created in Geneious Prime (v. 2019.0.3) and Adobe illustrator (v. 24.0.02).

**
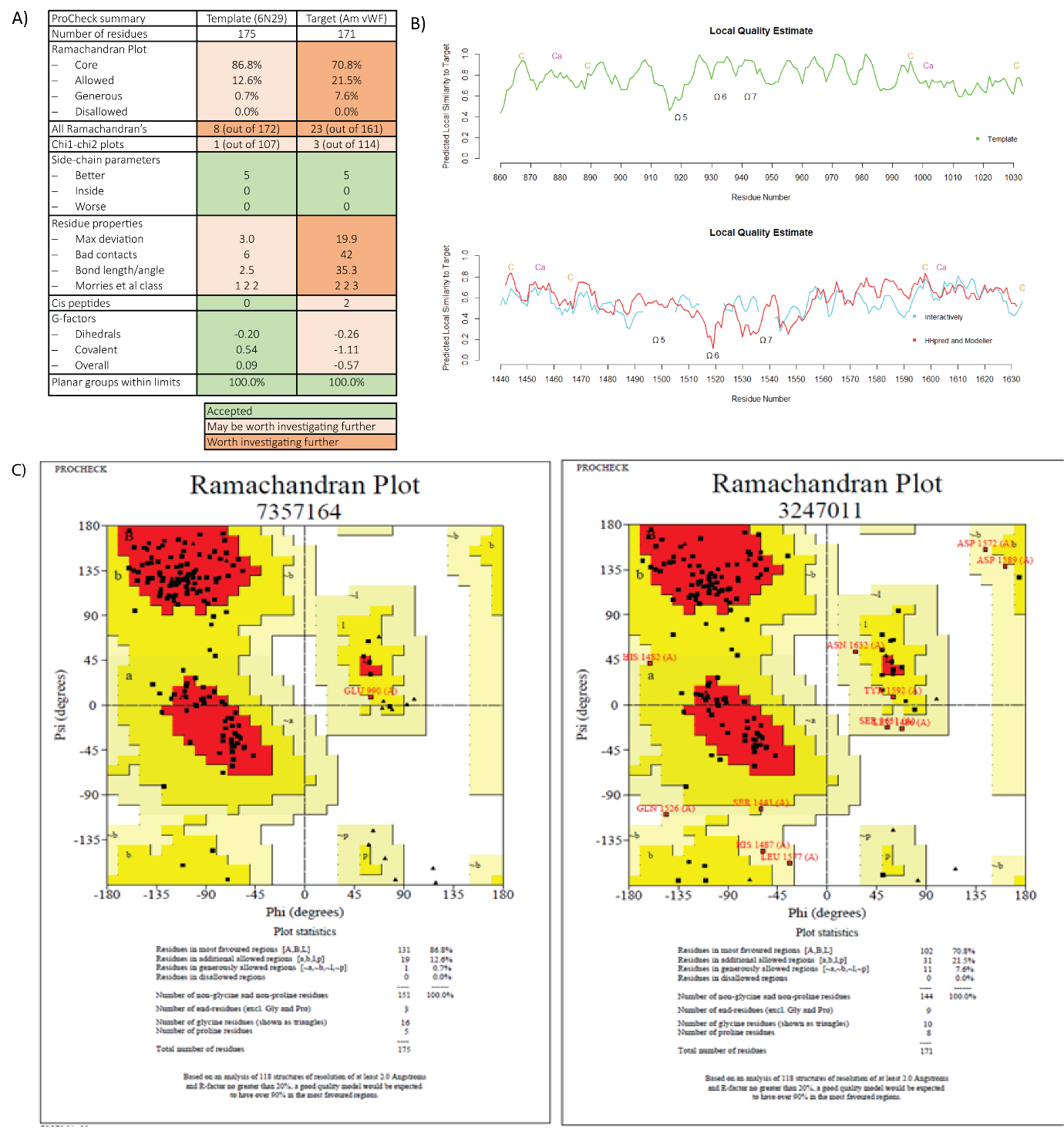
**

**Figure S3. ProCheck summary, local quality estimate and Ramachandran plots. A)** The ProCheck quality evaluations summarized and categorized by calculation results. The ideal residue values and standard deviation for any given model are derived from Morris et al. 1992.^1^ The max deviation, in residues properties, is calculated from the mean value of the residue-by-residue listing values (Fig. S4E) of the full-length structure. The number of bad contacts is defined as the non-bonded atoms at a distance of <= 2.6 Å. The bond length and angles are calculated in similar manner as the max deviation, but the ideal values are based on Engh and Huber 1991.^2^ The Morris et al. (1992) class summarizes the three above stereochemical parameters by assigning a number between 1 (best) to 4 (worst), indicating the overall quality of the model. **B)** Local QMEAN results are presented. The first plot is analysis of the template (green), while the second is analysis of the target modeled interactively (cyan) and automatically (red). The Ca^2+^-binding region (magenta Ca), the Cys residues forming the intra-chain disulfide bridges (orange, C) are in the higher quality region, while Ω5-7 (black) are in the lower quality region. The local score is calculated for each residue in the model and the average local score for the template is 0.93 ± 0.07, while the target average score is 0.40 ± 0.07 (cyan) and 0.44 ± 0.06 (red).  **C)** The Ramachandran plot produced by ProCheck. The plot on the left is the template (PDB ID: 6N29), while the target (honey bee vWF domain) is on the right. Below each plot, the statistic is presented.

**
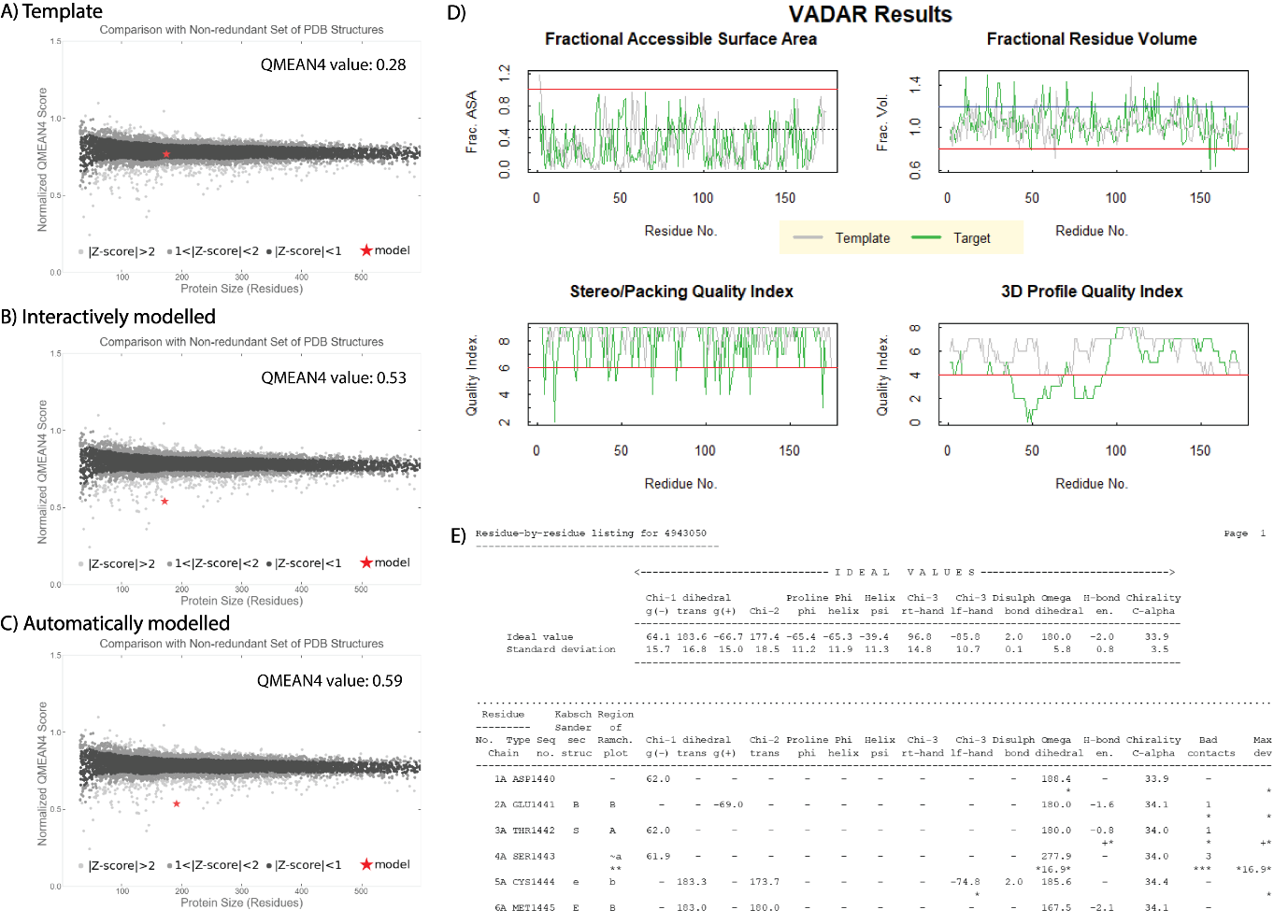
**

**Figure S4. Global quality estimate, VADAR plots and ProCheck residue listing. A-C)** The plots of the global QMEAN have the QMEAN4 scores for a set PDB structures plotted (gray dots) with the QMEAN4 score along the x-axis and the number of residues in the structures as long the y-axis. The global scores value QMEAN4 range from 0 to 1, where 1 is good. **A)** Analysis of the template (red star) and the QMEAN4 value is written on the plot. **B)** Analysis of the interactively homology modeled (red star) structure and **C)** The automatically homology modeled (red star) structure from MODELLER. **D)** Four different analyses were performed by VADAR, presented in one plot each, with the template (gray) compared to the target (green). **Plot 1**: a low fractional ASA score indicates a buried residue, while a score above 0.5 (dotted black line) indicates an exposed residue. A score above 1.0 (red line) indicates a problem in the structure. **Plot 2**: When a protein structure is efficiently packed the score should be around 1.0 ± 0.1. A score above 1.2 (blue line) or below 0.8 (red line) could indicate a poor refinement or identify cavities. **Plot 3**: Each residue is assigned a score between 0-3 (high is good quality) for three different measurements (torsion angle, omega angle and fractional volume). The total quality score for each residue can be from 0-9 and the threshold for a good quality is set to 6 (red line). **Plot 4**: Calculates the 3D quality of each residue based on its environment and gives a score between 0-9 (high is good quality), and the threshold for a good quality is set to 4 (red line). **E)** The Residue-by-Residue listing for ProCheck lists all residues in a structure and present all calculations for each. A short example is shown here for the first six residues in the target structure. Each value is compared to the ideal values which is noted on top. The deviating values are marked with * (one standard deviation) and + (half a standard deviation) sign. For example, the omega dihedral angle of residue S1443 is 16.9 standard deviation away from the ideal value, which is a result from the loop building of Ω1.

**
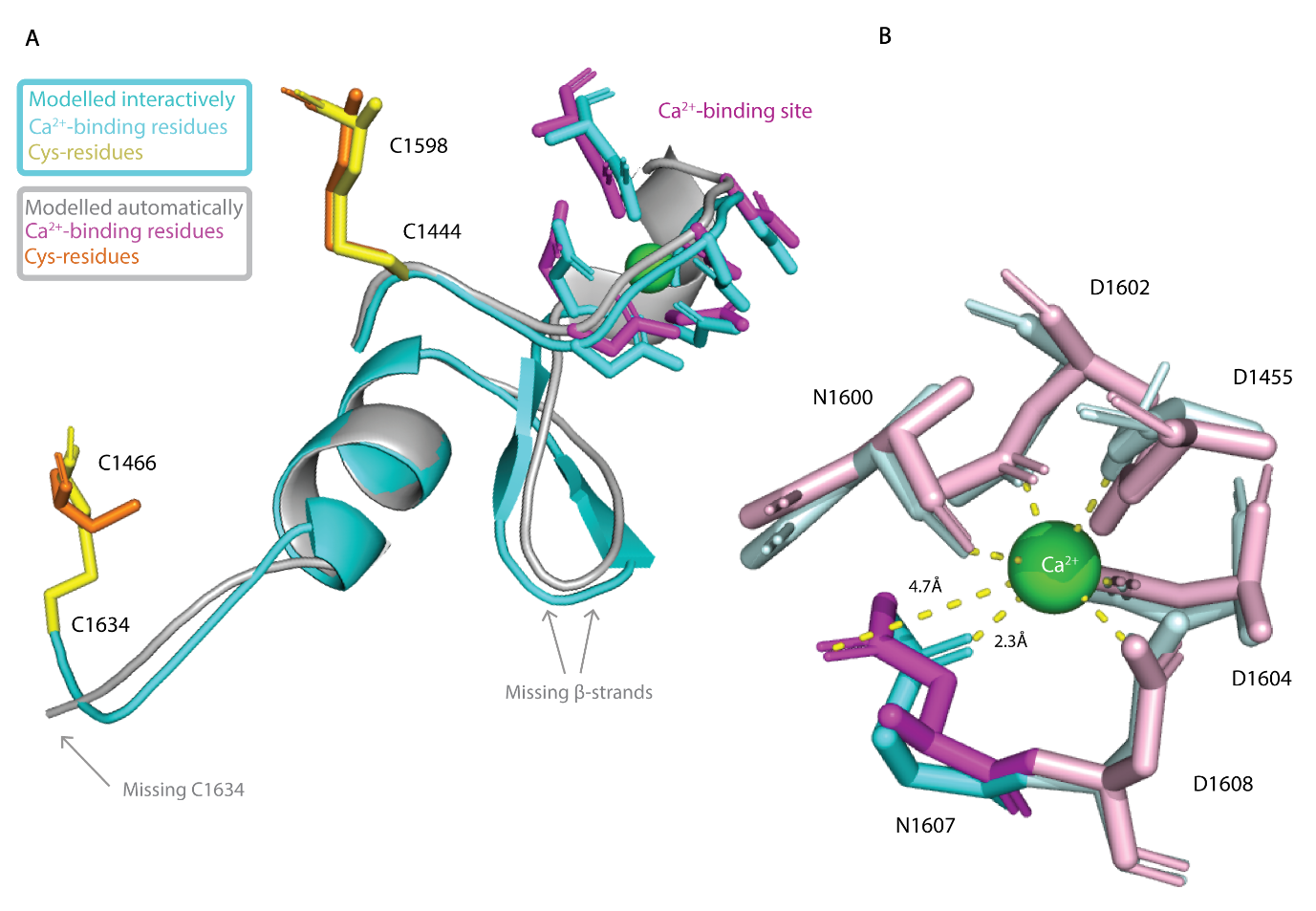
**

**Figure S5. Comparison of vWF homology models. A)** The sort region around the Ca^2+^-binding site (Ca^2+^-ion, green) is shown from the interactively modeled (cyan) structure and the automatically modeled (gray) structure. The Cys-residues (C1444, C1466, C1598 and C1634) and Ca^2+^-binding residues are shown as yellow/cyan (interactively) and orange/magenta (automatically) sticks. The missing C1634 and β-strands in the automatically modeled structure are shown (gray arrows). **B)** All the Ca^2+^-binding residues are in the same orientation in both models (light blue: interactively and light pink: automatically), except N1607. The interactions to the Ca^2+^-ion is shown as yellow dotted lines and measured (Å) for N1607.

**
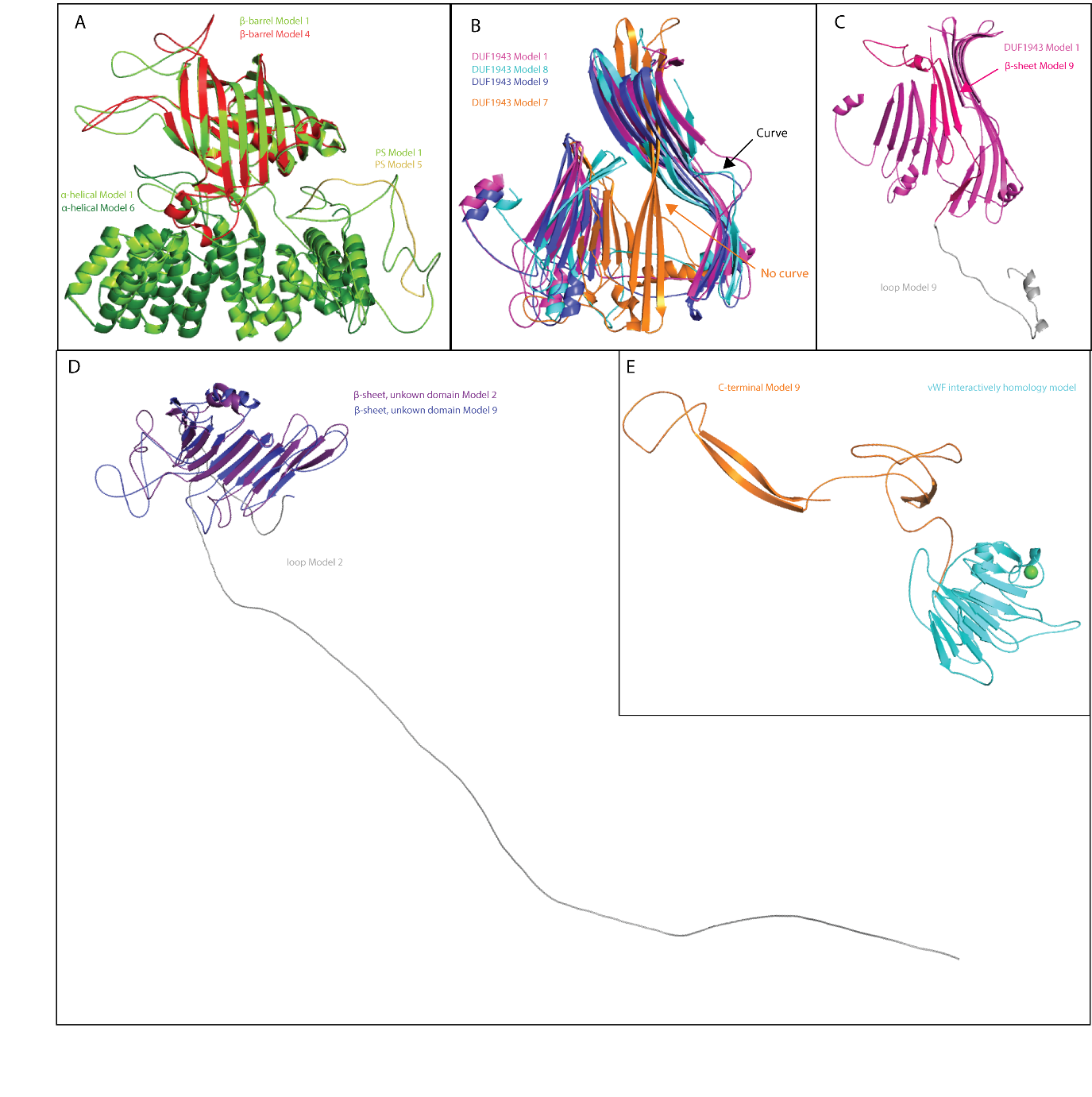
**

**Figure S6. Comparison of homology models from MODELLER and RaptorX.** **A)** The N-terminal domain: Model 1 (green) aligned with Model 4 (red), 5 (yellow) and 6 (forest green). **B) T**he DUF1943 domain: Model 1 (magenta) aligned with Model 8 (cyan), Model 7 (orange) and Model 9 (blue). The identified curve in the longer β-sheet in Model 1, 8 and 9 and the missing curve in Model 7 is marked with arrows. **C)** The DUF1943 domain Model 1 (magenta), the downstream region residue 1060 to 1140 of Model 9 (hot pink) and the loop region (gray). **D)** The undetermined domain: Model 2 (purple) aligned with Model 9 (blue), with the long loop region (gray). **E)** The interactively homology model of vWF domain (cyan) with the C-terminal region from Model 9 (orange).

**
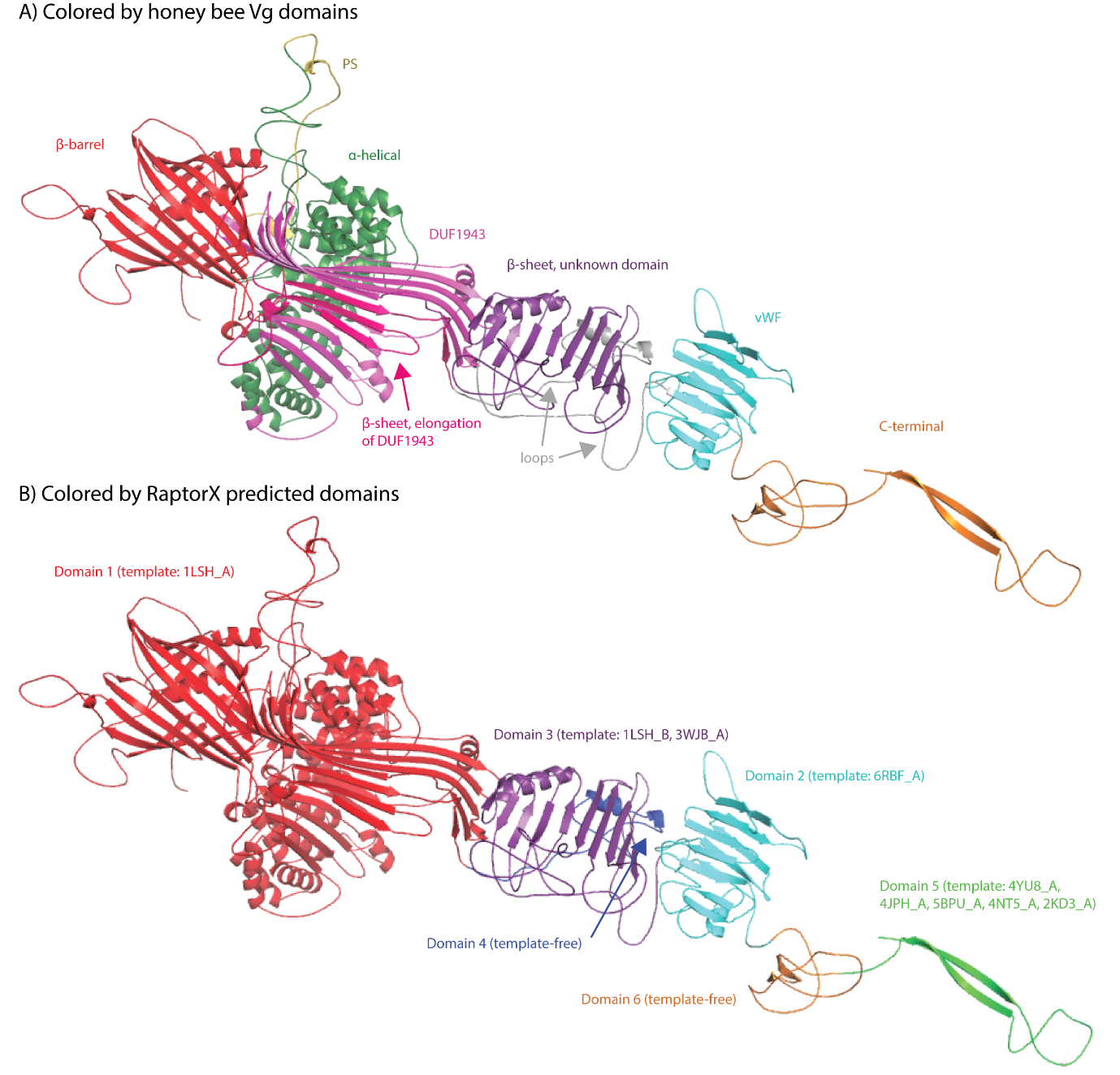
**

**Figure S7. RaptorX structural prediction of full-length honey bee vitellogenin.** **A)** The β-barrel subdomain (red), the polyserine linker (yellow), the α-helical subdomain (forest green), the DUF1943 domain (magenta), elongation of the DUF1943 domain (hot pink arrow), the undetermined structural region (purple), the vWF domain (cyan) and the C-terminal region (orange) are generated as one full-length model. The two loop regions (gray arrows) are also predicted. **B)** Domain 1 to 6 from Table S7 are colored red, cyan, purple, blue, green and orange, respectively, and if templates was used, the PDB ID is written in parenthesis.

**
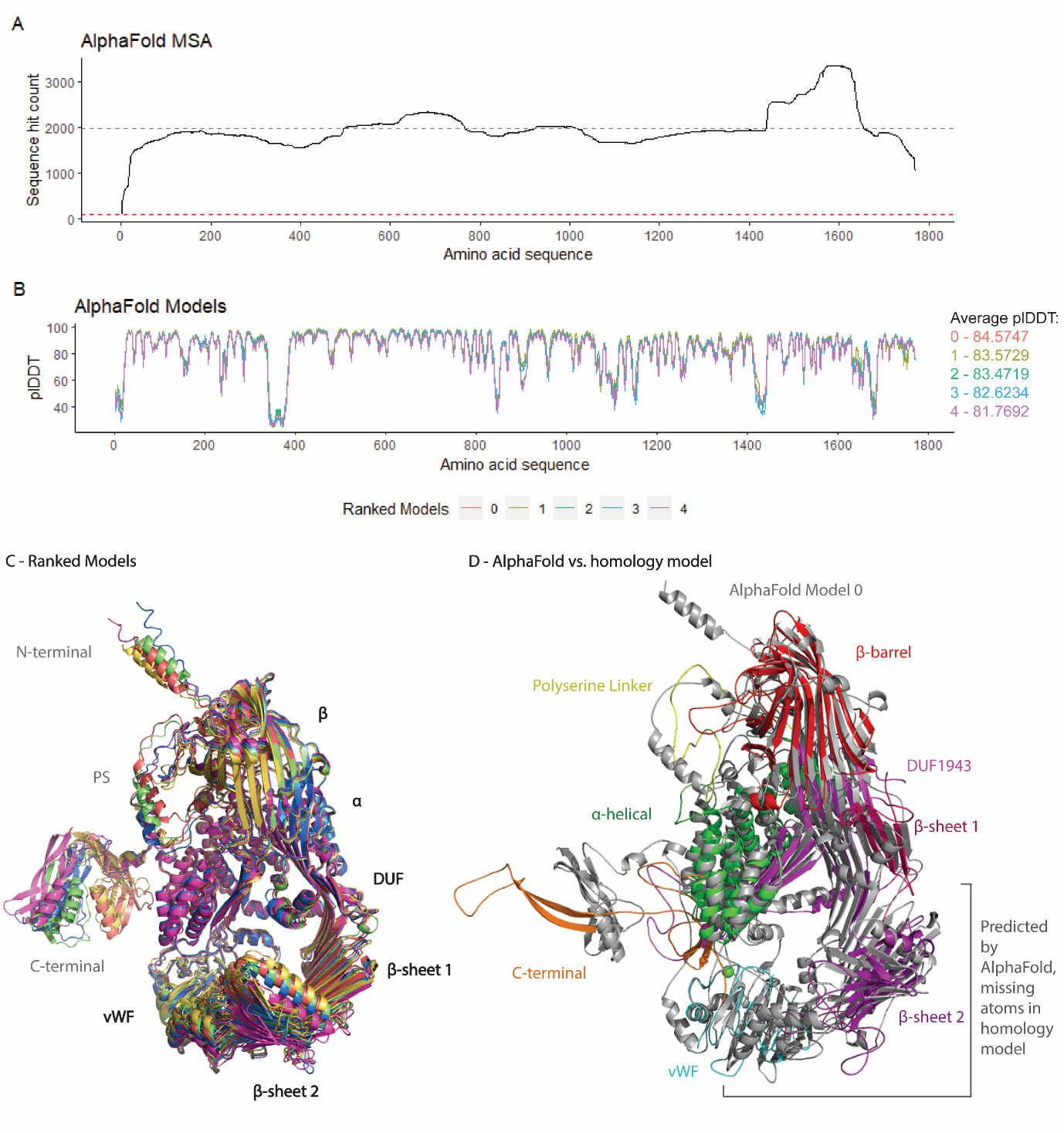
**

**Figure S8. AlphaFold output. A)** The number of sequence hits in the MSA produced by AlphaFold, is plotted per residue. The average number of hits per residue (gray dotted line), and the threshold at 100 sequence per residue (red dotted line) is marked. **B)** The plDDT score for the five outputted models by AlphaFold is plotted per residue, and the average plDDT score per model is listed to the right, which produces the rank from 0 (best) to 4 (worst). **C)** The ranked models are aligned, colored by the same coloring scheme in panel B, and the consistently folded domains (β-barrel (β), α-helical (α), DUF1943 (DUF), β-sheet 1 (β1), β-sheet 2 (β2) and vWF domain (vWF)) are labeled in bold letters, while the more variable domains (N-terminal, polyserine linker (PS) and C-terminal) are labeled in grey letters. **D)** The final homology model domains (β-barrel (red), polyserine linker (yellow), α-helical (green), DUF1943 (magenta), β-sheet 1 (hotpink), β-sheet 2 (purple), vWF (cyan, Ca^2+^-ion shown as green sphere) and C-terminal domain (orange) is aligned to their respective domains in the top ranked AlphaFold prediction (grey). The grey brackets to the lower right indicate the region where AlphaFold have predicted a fold for the main missing atoms in the homology model.

**
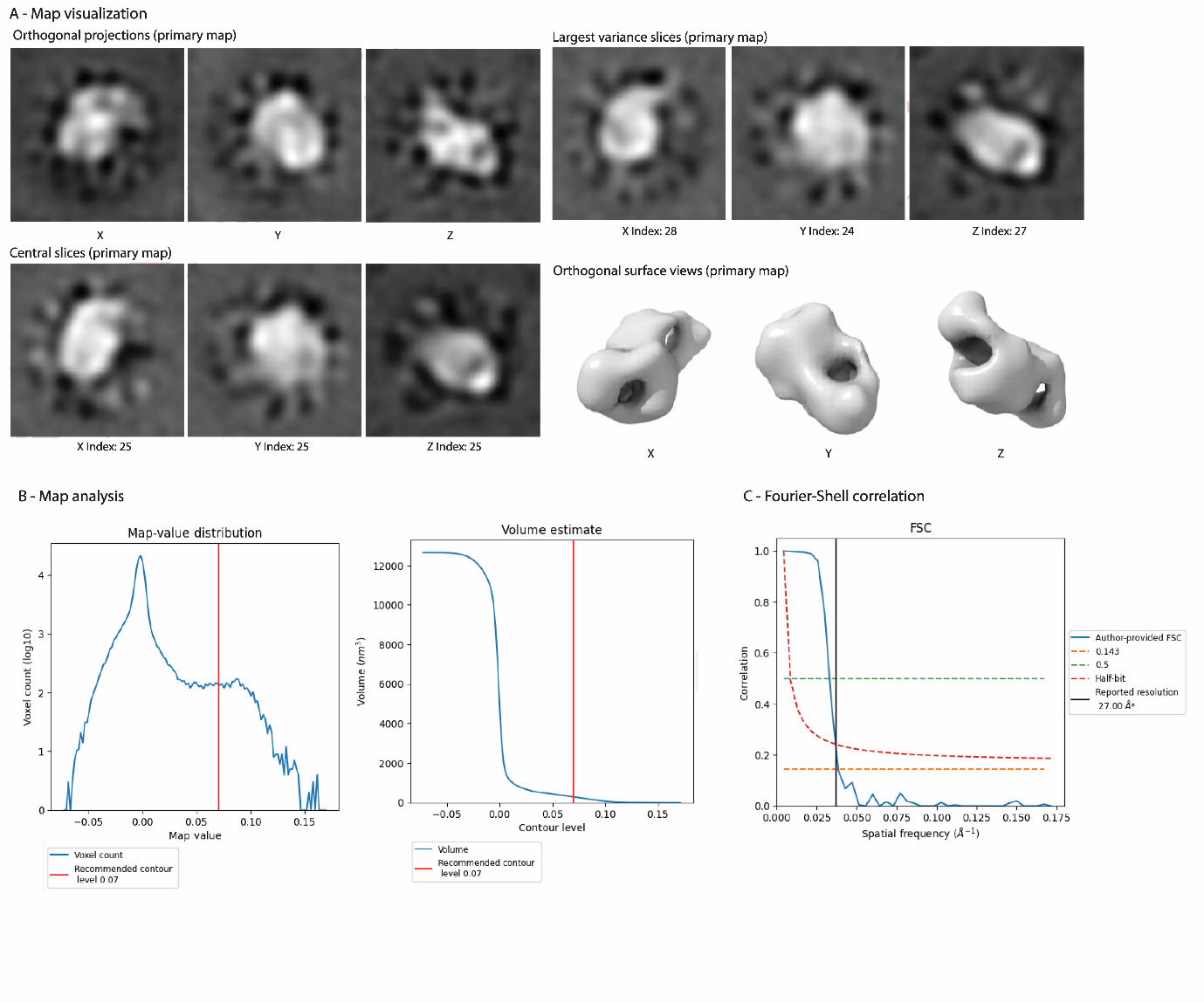
Figure S9. EM map validation. A)** Map visualization to allow visual inspection of the internal detail of the map and identification of artifacts. The primary map, central slices of the map and largest variance of the map is shown in three orthogonal directions. The 3D surface view of the primary map at recommended contour level 0.07. **B)** Statistical analysis of the map. In the first graph the map-value distributions is plotted in 128 intervals along the x-axis, and the y-axis is logarithmic. The spike around 0 indicate that the volume has been masked. The second graph shows how the enclosed volume varies with the contour level. The volume at the recommended contour (red line) is 289 nm3; this corresponds to an approximate mass of 261 kDa. **C)** The provided Fourier-Shell Correlation (blue) is plotted together with the reported resolution, (black line, *Reported resolution corresponds to spatial frequency of 0.037 Å^-1^). A curve is displayed for the half-bit criterion (dashed red), in addition to lines showing the 0.143 gold standard cut-off (dashed orange line) and 0.5 cut-off (green dotted line). All the graphs are assembled from the EmDataBank map validation report (copy included).

**
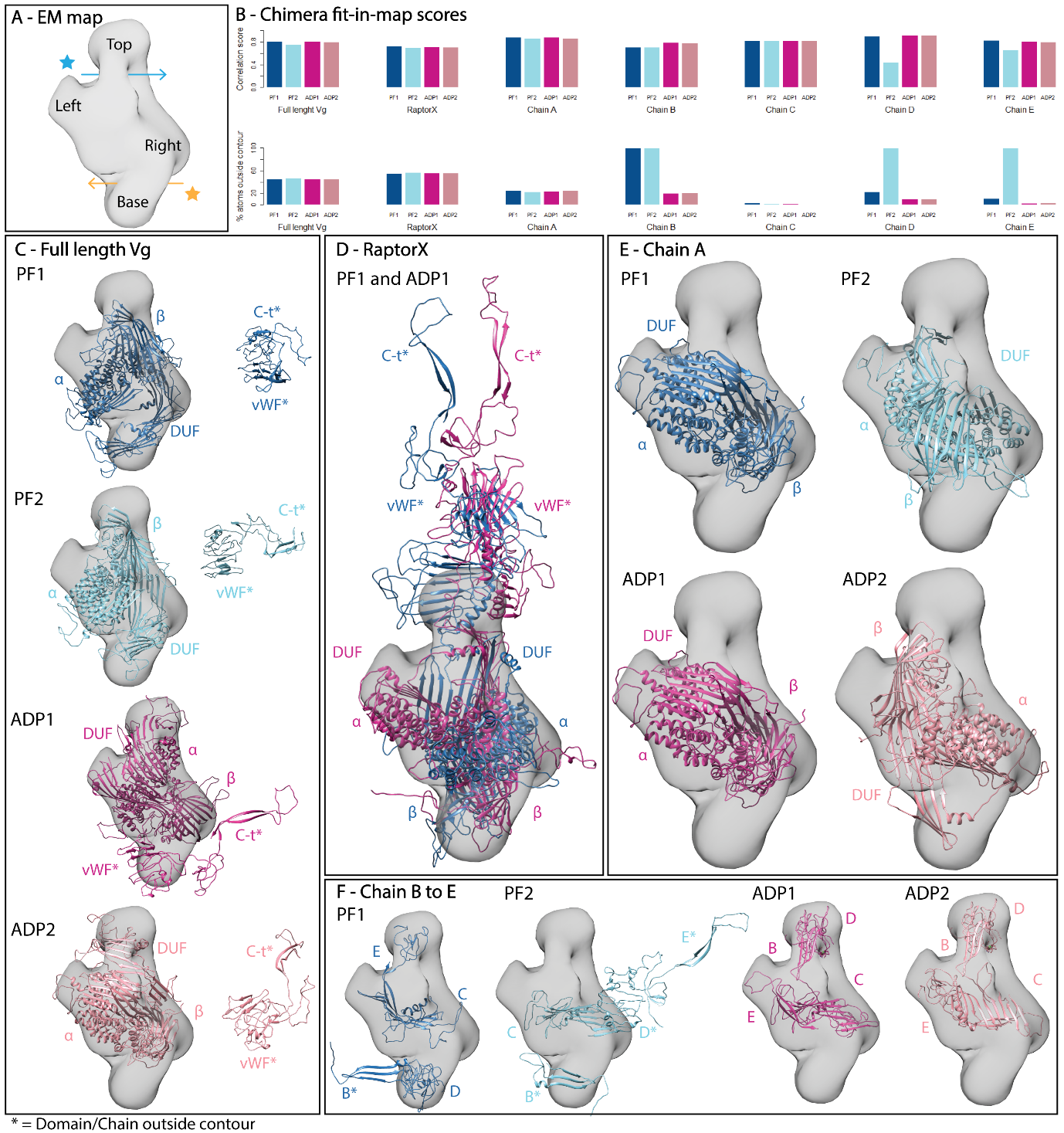
Figure S10. Rigid-body fitting for honey bee vitellogenin homology models**. **A)** The EM map is shown as a gray surface. The distinct cavity creases are marked with stars and arrows, upper cavity (blue) and lower cavity (yellow). The four curves in the surface are labeled (top, base, left and right). **B)** The correlation score and precent of atoms outside the contour calculated by Chimera was plotted for each fit from PowerFit (PF, blue) and ADP_EM (ADP, pink), and ranked according to the correlation score (dark color: highest score, light color: second highest score). **C-E)** The fits from the full-length homology model, RaptorX and chain A is presented inside the EM map, with the same coloring scheme as in panel B. The β-barrel (β), α-helical (α), DUF1943 (DUF), vWF and C-terminal (C-t) domains are labeled. If the domain is outside of the contour it is noted by a “*”-mark. **F)** The fits of chain B to E separately with the same coloring scheme as in panel B, but they are labeled according to chains and not domains.

**
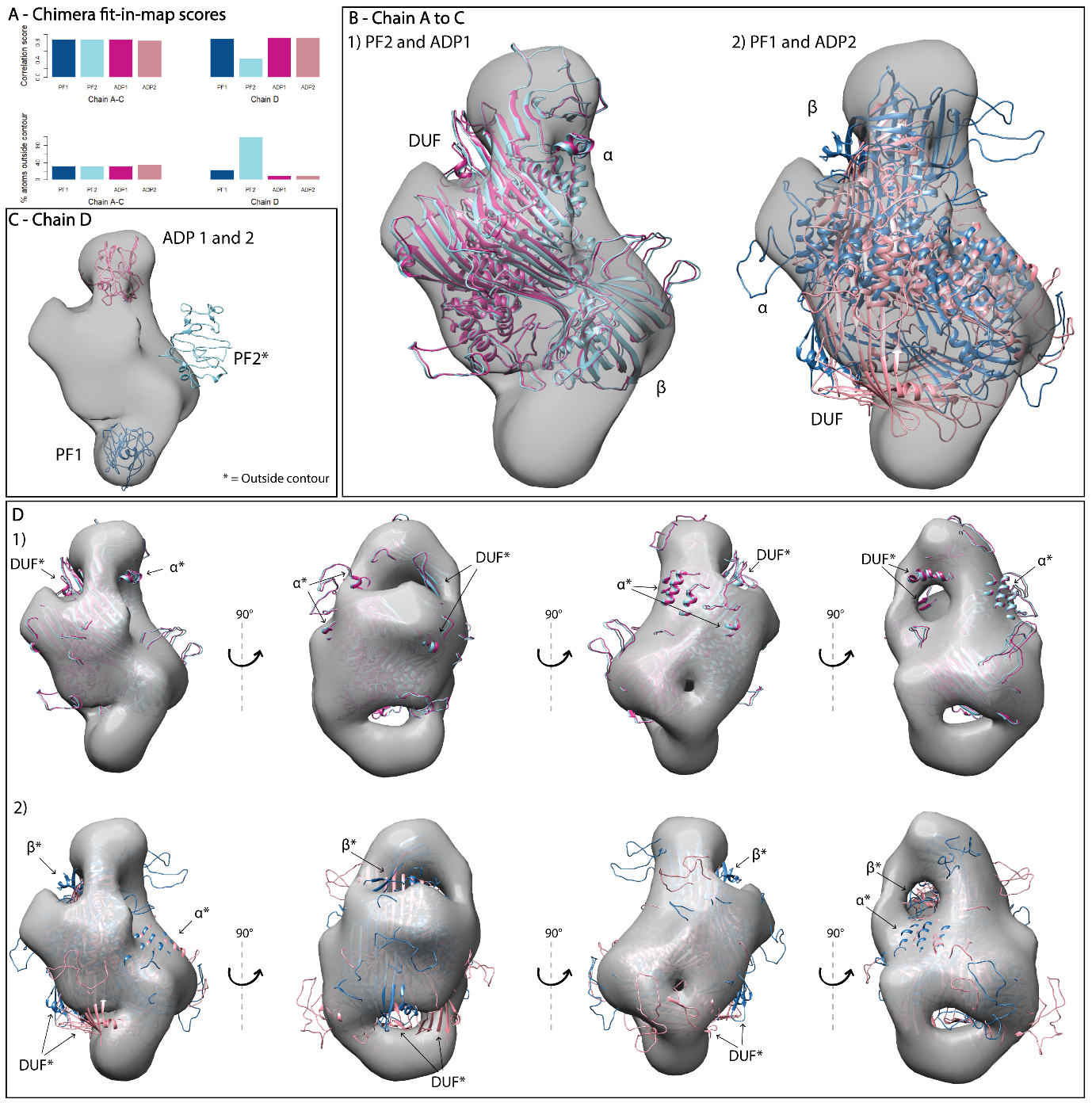
**

**Figure S11. Rigid-body fitting of chain A to C and D. A)** The correlation score and precent of atoms outside the contour calculated by Chimera was plotted for each fit from PowerFit (PF, blue) and ADP_EM (ADP, pink), and ranked according to the correlation score (dark color: highest score, light color: second highest score). **B)** The EM map are shown as a transparent surface, and the fits of chain A to C from PF and ADP are shown as cartoons and colored by method and scores (dark blue: PF1, light blue: PF2, dark pink: ADP1, light pink: ADP2). The β-barrel (β), α-helical (α) and DUF1943 (DUF) domains are labeled. **C)** The EM map and the fits of chain D is shown in same coloring scheme as in panel B. The label is marked with “*” if the fit is outside the contour of the EM map. **D)** The EM map are shown as a surface, less transparent than in panel B, with the fits of chain A to C (1: PF2 and ADP1, 2: PF1 and ADP2) in the same coloring scheme as in panel B. The EM map is shown at four different angels, and arrows points to secondary structure elements from β, α or DUF domain which are outside the contour of the EM map.

**
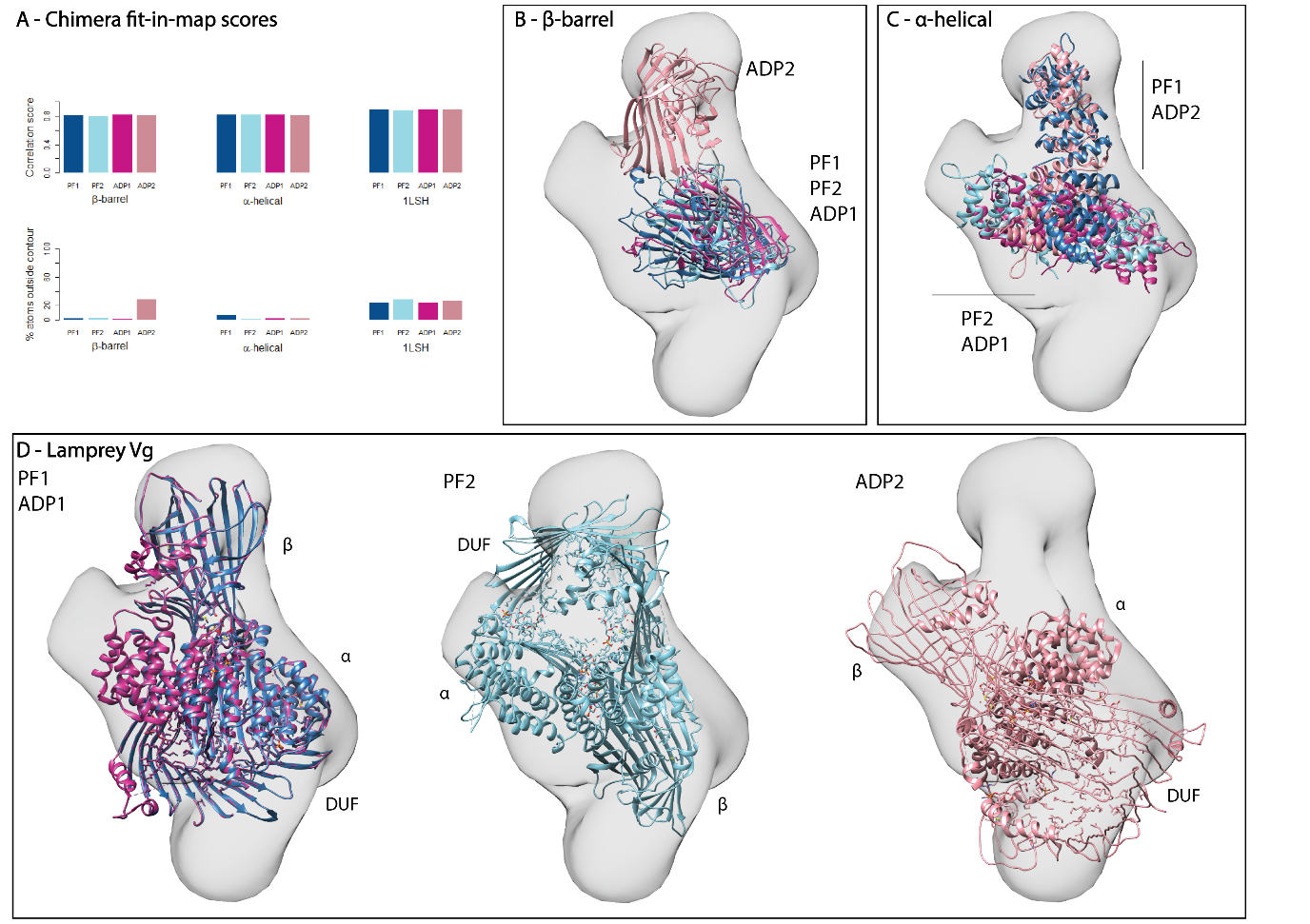
**

**Figure S12. Rigid-body fitting for previously published homology models and a distant homologue. A)** The same plot as in Fig. S10 for the β-barrel and α-helical subdomains, and the crystal structure of lamprey Vg (1LSH). **B-D)** Same presentation and coloring scheme as in Fig. S10C-S10F.

### References

1. Morris AL, MacArthur MW, Hutchinson EG, Thornton JM. 1992. Stereochemical quality of protein structure coordinates. Proteins. 12(4):345-364.

2. Engh RA, Huber R. 1991. Accurate bond and angle parameters for x-ray protein structure refinement. Acta Crystallographica Section A. 47(4):392-400.
