# Supplementary material for: Structure prediction of honey bee vitellogenin: a multi‐domain protein important for insect immunity
Source: FEBS Open Bio. 2021 Oct 31;12(1):51–70. doi: 10.1002/2211-5463.13316 (PMC8727950; doi:10.1002/2211-5463.13316)
Supplement: Supplementary file 2 — Appendix S1. wwPDB EM Validation Summary Report. [file FEB4-12-51-s001.pdf]

wwPDB EM Validation Summary Report (i)

Feb 23, 2021 – 11:46 AM EST

EMDB ID	:	EMD-22113
Title	:	Honey bee vitellogenin
Deposited on	:	2020-06-03
Resolution	:	27.00 Å(reported)

This is a wwPDB EM Validation Summary Report.

This report is produced by the wwPDB biocuration pipeline after annotation of the structure.

We welcome your comments at *validation@mail.wwpdb.org*
A user guide is available at
https://www.wwpdb.org/validation/2017/EMMapValidationReportHelp
with specific help available everywhere you see the (i) symbol.

CONFIDENTIAL VALIDATION REPORT

The following versions of software and data (see references (i)) were used in the production of this report:

EMDB validation analysis	:	0.0.0.dev61
Validation Pipeline (wwPDB-VP)	:	2.17.1

1 Experimental information (i)

Property	Value	Source
EM reconstruction method	SINGLE PARTICLE	Depositor
Imposed symmetry	POINT, Not provided	
Number of particles used	15000	Depositor
Resolution determination method	Not provided	
CTF correction method	PHASE FLIPPING AND AMPLITUDE CORRECTION	Depositor
Microscope	JEOL 1230	Depositor
Voltage (kV)	100	Depositor
Electron dose $(e^-/\text{Å}^2)$	25	Depositor
Minimum defocus (nm)	1500	Depositor
Maximum defocus (nm)	3000	Depositor
Magnification	Not provided	
Image detector	TVIPS TEMCAM-F415 (4k x 4k)	Depositor
Maximum map value	0.171	Depositor
Minimum map value	-0.072	Depositor
Average map value	0.001	Depositor
Map value standard deviation	0.018	Depositor
Recommended contour level	0.07	Depositor
Map size (Å)	233.0, 233.0, 233.0	wwPDB
Map dimensions	50, 50, 50	wwPDB
Map angles (°)	90.0, 90.0, 90.0	wwPDB
Pixel spacing (Å)	4.66, 4.66, 4.66	Depositor

CONFIDENTIAL VALIDATION REPORT

2 Map visualisation (i)

This section contains visualisations of the EMDB entry EMD-22113. These allow visual inspection of the internal detail of the map and identification of artifacts.

No raw map or half-maps were deposited for this entry and therefore no images, graphs, etc. pertaining to the raw map can be shown.

2.1 Orthogonal projections (i)

2.1.1 Primary map

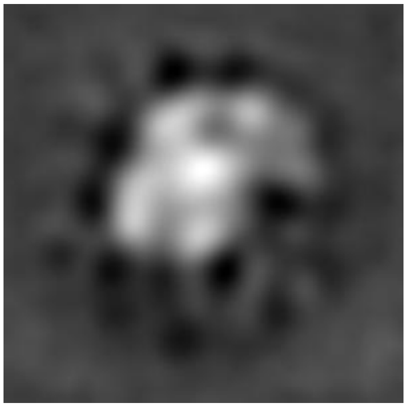

X

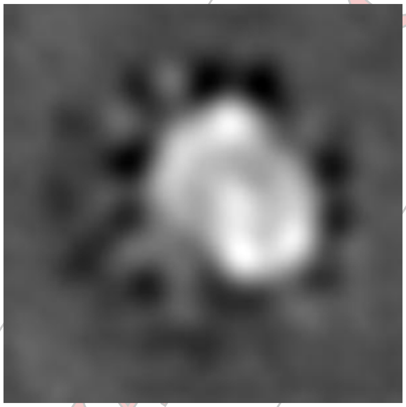

Y

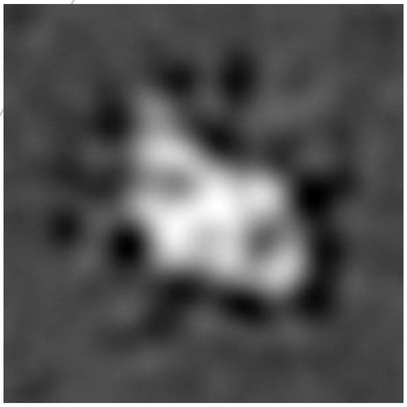

Z

The images above show the map projected in three orthogonal directions.

2.2 Central slices (i)

2.2.1 Primary map

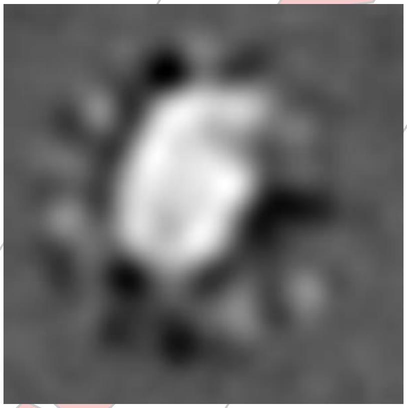

X Index: 25

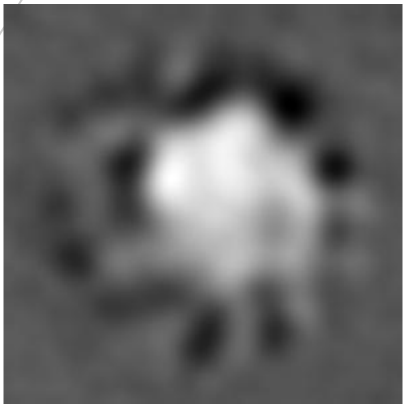

Y Index: 25

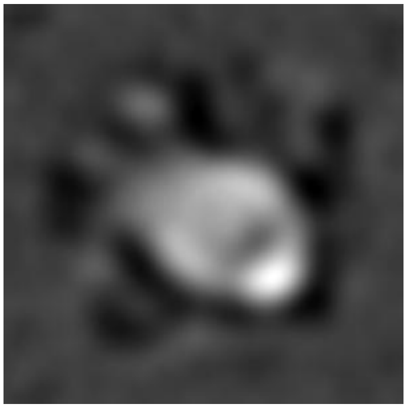

Z Index: 25

CONFIDENTIAL VALIDATION REPORT

The images above show central slices of the map in three orthogonal directions.

2.3 Largest variance slices (i)

2.3.1 Primary map

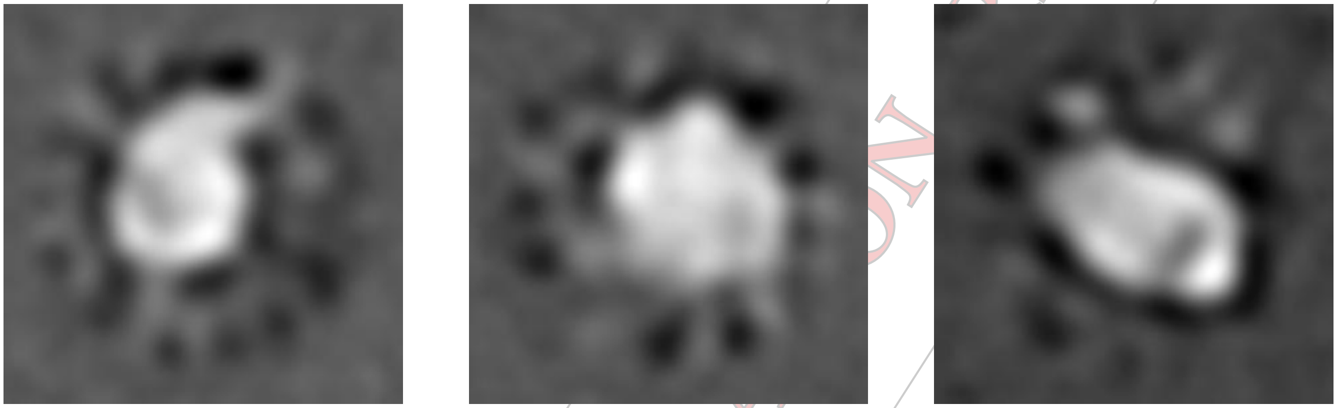

X Index: 28 Y Index: 24 Z Index: 27

The images above show the largest variance slices of the map in three orthogonal directions.

2.4 Orthogonal surface views (i)

2.4.1 Primary map

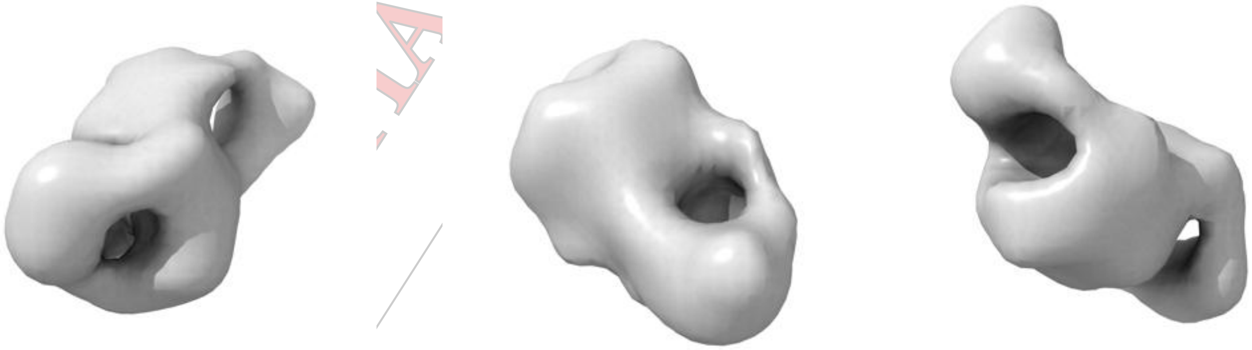

X Y Z

The images above show the 3D surface view of the map at the recommended contour level 0.07. These images, in conjunction with the slice images, may facilitate assessment of whether an appropriate contour level has been provided.

CONFIDENTIAL VALIDATION REPORT

2.5 Mask visualisation ⓘ

This section was not generated. No masks/segmentation were deposited.

CONFIDENTIAL VALIDATION REPORT

3 Map analysis (i)

This section contains the results of statistical analysis of the map.

3.1 Map-value distribution (i)

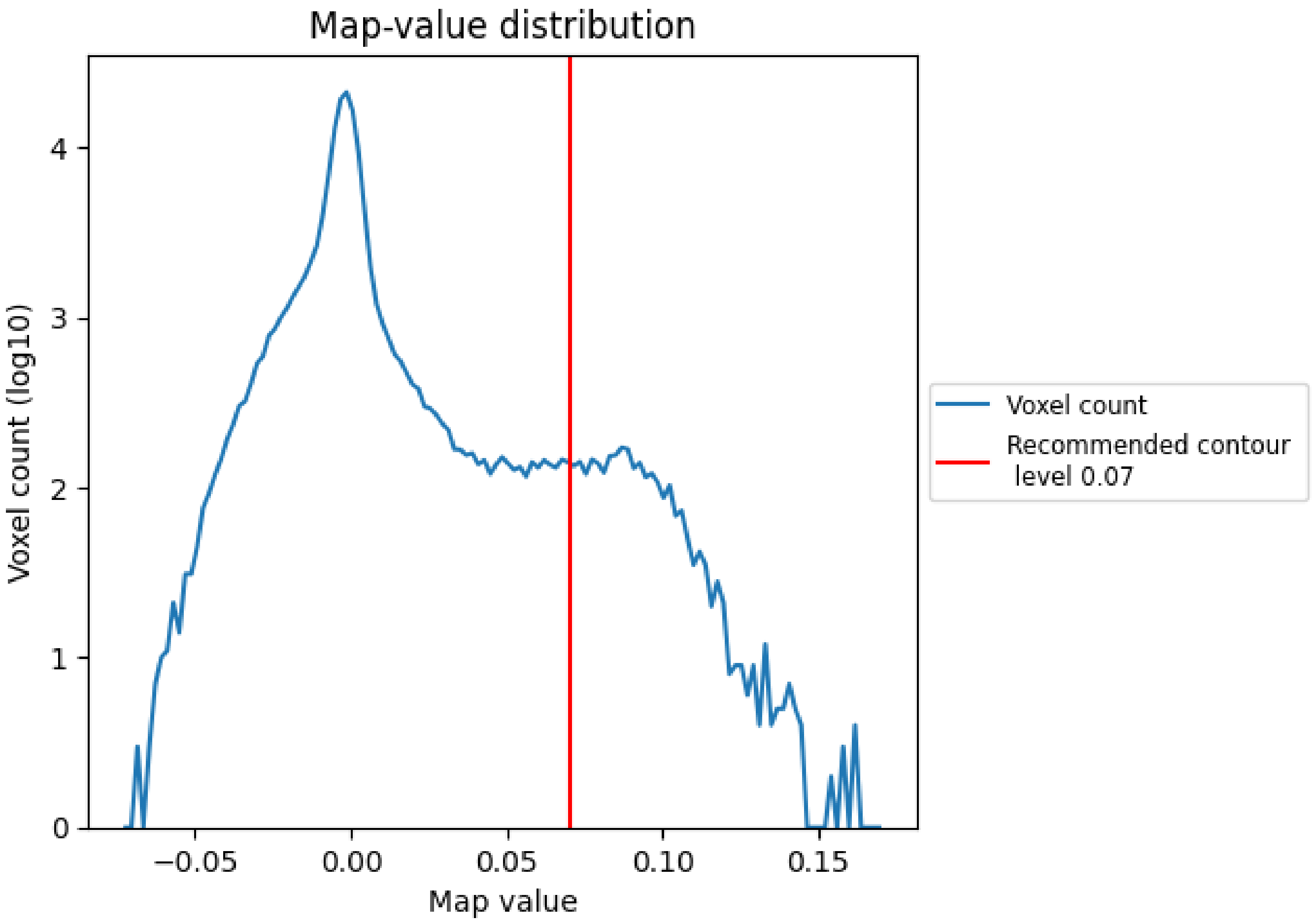

The map-value distribution is plotted in 128 intervals along the x-axis. The y-axis is logarithmic. A spike in this graph at zero usually indicates that the volume has been masked.

CONFIDENTIAL REPORT

3.2 Volume estimate (i)

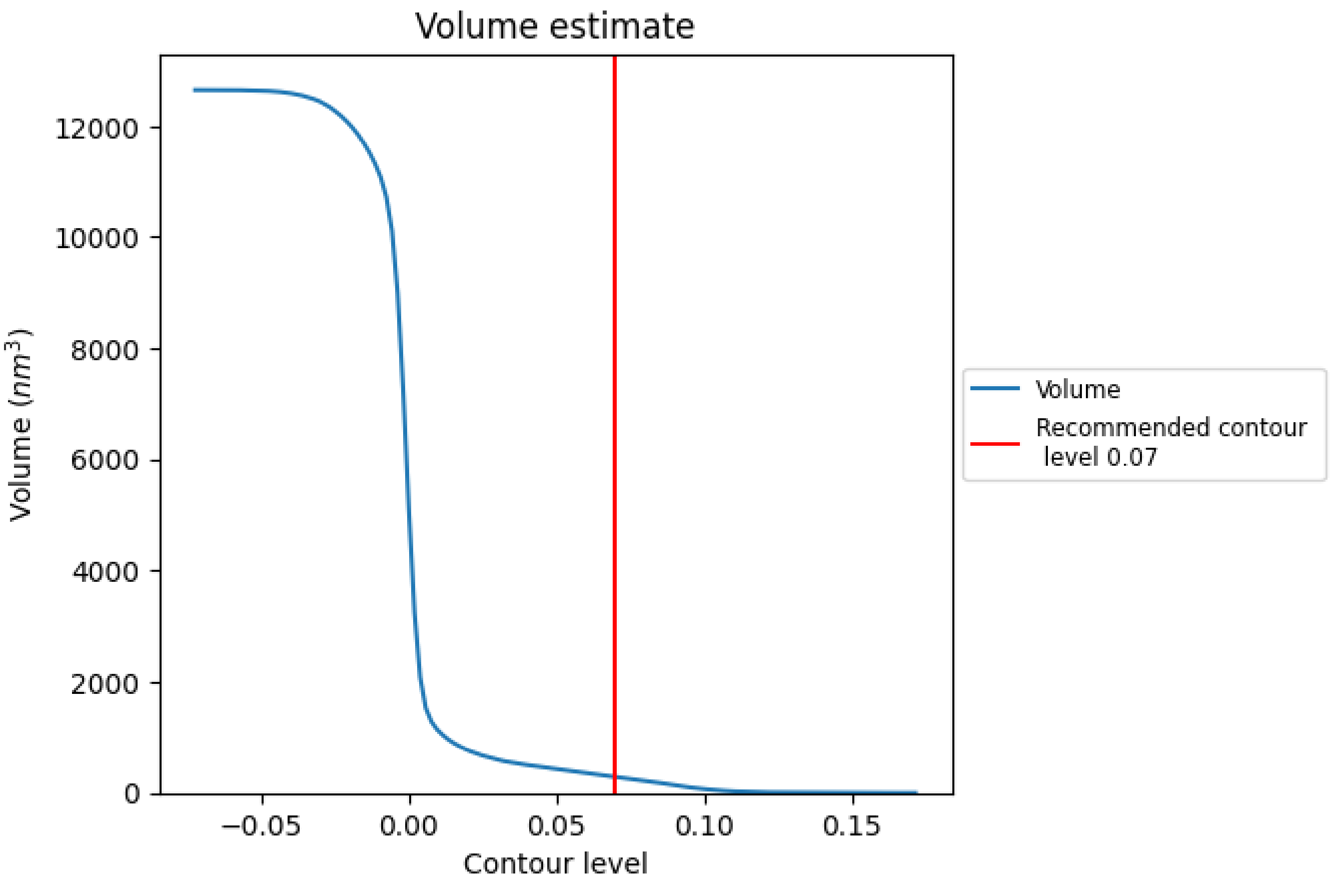

The volume at the recommended contour level is 289 nm^3; this corresponds to an approximate mass of 261 kDa.

The volume estimate graph shows how the enclosed volume varies with the contour level. The recommended contour level is shown as a vertical line and the intersection between the line and the curve gives the volume of the enclosed surface at the given level.

CONFIDENTIAL REPORT

3.3 Rotationally averaged power spectrum ⓘ

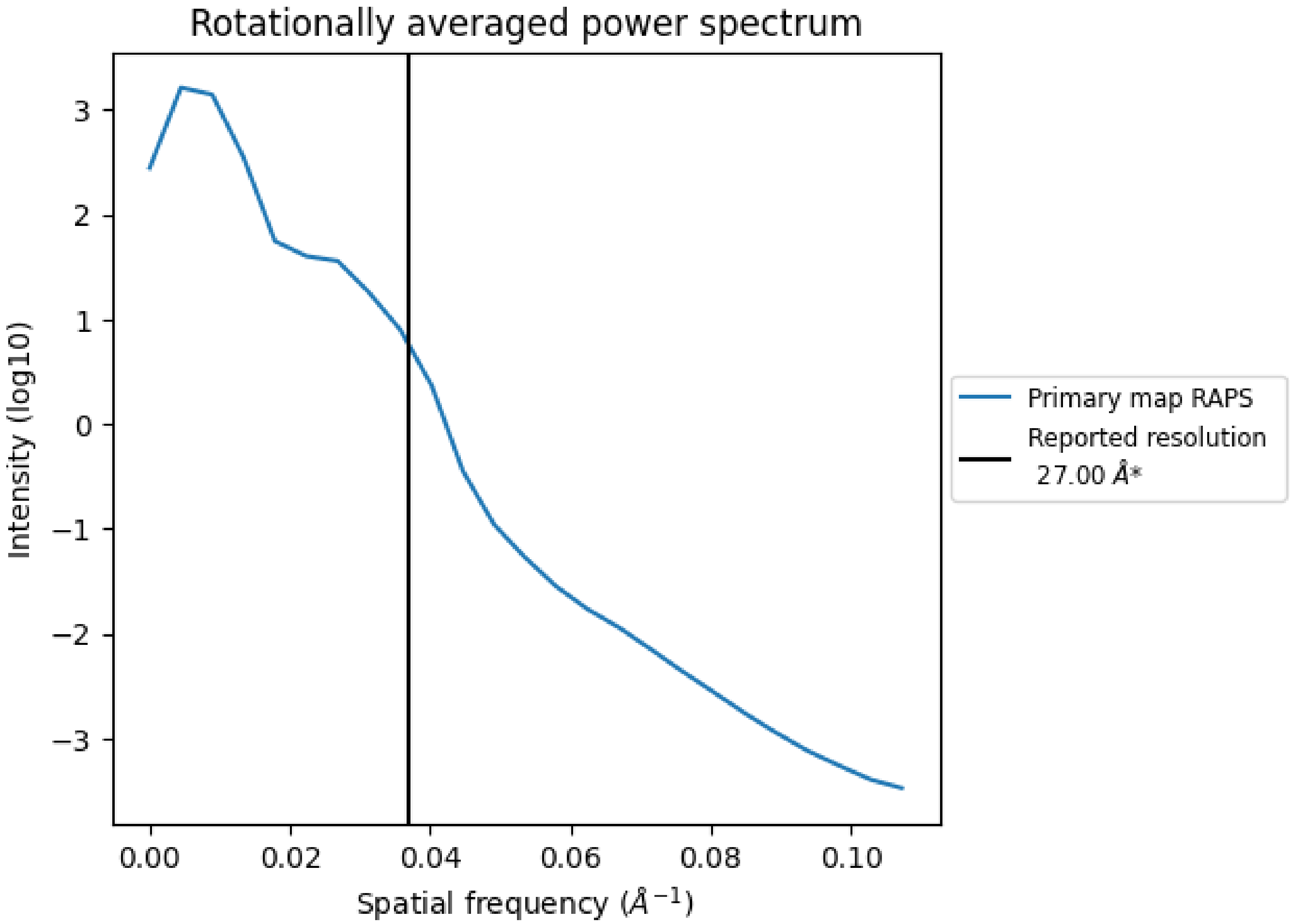

*Reported resolution corresponds to spatial frequency of 0.037 Å^{-1}

CONFIDENTIAL REPORT

4 Fourier-Shell correlation (i)

Fourier-Shell Correlation (FSC) is the most commonly used method to estimate the resolution of single-particle and subtomogram-averaged maps. The shape of the curve depends on the imposed symmetry, mask and whether or not the two 3D reconstructions used were processed from a common reference. The reported resolution is shown as a black line. A curve is displayed for the half-bit criterion in addition to lines showing the 0.143 gold standard cut-off and 0.5 cut-off.

4.1 FSC (i)

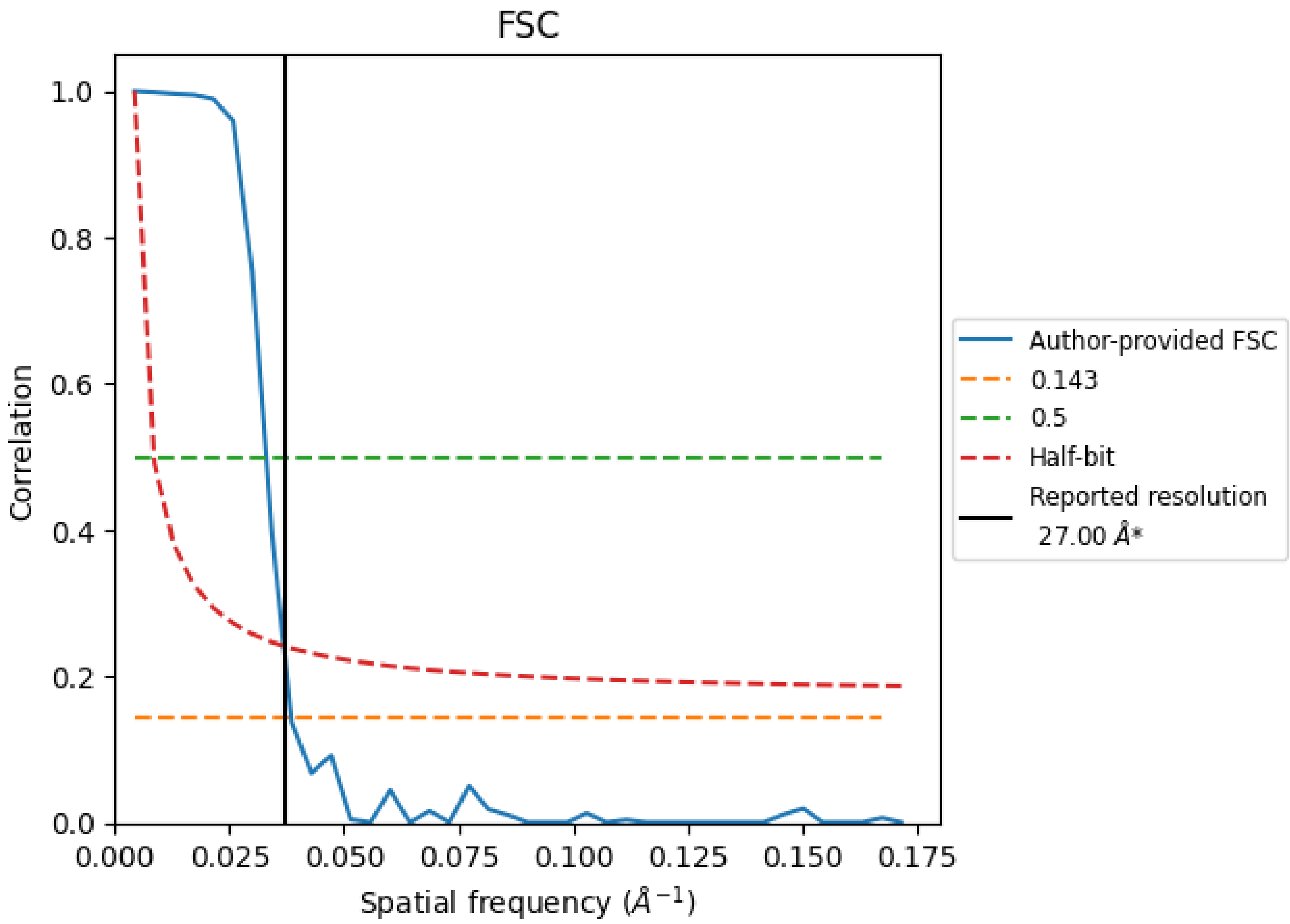

*Reported resolution corresponds to spatial frequency of 0.037 $Å^{-1}$

CONFIDENTIAL REPORT

4.2 Resolution estimates (i)

Resolution estimate (Å)	Estimation criterion (FSC cut-off)		
	0.143	0.5	Half-bit
Reported by author	-	-	-
Author-provided FSC curve	29.24	34.72	30.67
Calculated*	-	-	-

*Resolution estimate based on FSC curve calculated by comparison of deposited half-maps.

CONFIDENTIAL VALIDATION REPORT
